# Baxdrostat versus osilodrostat: steroid biosynthesis in human adrenocortical cells

**DOI:** 10.1530/EC-25-0807

**Published:** 2026-07-15

**Authors:** Yuki Taki, Takashi Kono, Ikki Sakuma, Naoko Hashimoto, Masataka Yokoyama, Eiryo Kawakami, Takashi Miki, Hironobu Sasano, Yasuhiro Nakamura, Tomoaki Tanaka

**Affiliations:** ^1^ Department of Molecular Diagnosis, Chiba University Graduate School of Medicine, Chiba, Japan; ^2^ Research Institute of Disaster Medicine, Chiba University, Chiba, Japan; ^3^ Department of Medical Physiology, Graduate School of Medicine, Chiba University, Chiba, Japan; ^4^ Department of Artificial Intelligence Medicine, Graduate School of Medicine, Chiba University, Chiba, Japan; ^5^ Department of Pathology, Tohoku University School of Medicine, Sendai, Japan; ^6^ Division of Pathology, Faculty of Medicine, Tohoku Medical and Pharmaceutical University, Sendai, Japan

**Keywords:** aldosterone synthase inhibitor, baxdrostat, osilodrostat, primary human adrenal cells, LC–MS/MS, resistant hypertension

## Abstract

To compare the functional selectivity of baxdrostat versus osilodrostat in primary human adrenocortical cells, focusing on aldosterone suppression while preserving cortisol biosynthesis, we conducted an experimental *ex vivo* study using primary cells derived from aldosterone-producing adenomas, cortisol-producing tumors, and normal adrenal cortex. Cells were stimulated with the adrenocorticotropic hormone (10 nM) and exposed for 72 h to baxdrostat, osilodrostat, or metyrapone (0–10 μM). Outcomes included geometric mean (GM) IC_50_ values for aldosterone and cortisol, LC–MS/MS steroid profiling, and molecular docking to CYP11B2/CYP11B1. In aldosterone-producing adenoma cells, baxdrostat selectively inhibited aldosterone (GM IC_50_ = 0.041 μM) and did not achieve 50% cortisol suppression in cortisol-producing tumor or normal adrenal cortex within 0–10 μM. Osilodrostat inhibited aldosterone (GM IC_50_ = 0.0022 μM) and cortisol (GM IC_50_ = 0.196 μM). Steroid profiling showed a modest, selective rise in 11-deoxycorticosterone with baxdrostat (+3.76-fold), whereas osilodrostat produced larger increases in 11-deoxycorticosterone (+25.4-fold) together with androgen elevations. Docking supported the mechanism: baxdrostat showed stronger predicted binding to CYP11B2 (−11.5 kcal/mol) than osilodrostat (−8.7 kcal/mol) with limited engagement of CYP11B1. Baxdrostat demonstrated greater functional selectivity for CYP11B2, with minimal cortisol axis impact and smaller upstream precursor accumulation than osilodrostat. These human-tissue data inform dose selection and safety monitoring strategies for selective aldosterone synthase inhibition in aldosterone-mediated and treatment-resistant hypertension.

## Introduction

Aldosterone excess contributes to hypertension and adverse cardiovascular outcomes through mineralocorticoid receptor (MR)-mediated sodium retention and nongenomic signaling (1, 2). MR antagonists (MRAs) improve blood pressure and target-organ damage, yet their long-term utility is constrained by safety and tolerability (3, 4). MRAs may cause hyperkalemia, particularly in patients with chronic kidney disease or diabetes, and spironolactone also causes antiandrogenic effects (e.g. gynecomastia) (5, 6). Moreover, MRAs do not address the source of aldosterone excess and may permit compensatory increases in aldosterone production (7, 8). To mitigate off-target hormonal effects while preserving efficacy, selective MRAs, such as eplerenone and the newer nonsteroidal esaxerenone, were developed; however, dose-limiting hyperkalemia can persist in susceptible patients, leaving a subset uncontrolled (9, 10).

Treatment-resistant hypertension (TRH) is characterized by uncontrolled blood pressure despite the use of ≥3 antihypertensive drug classes at maximally tolerated doses, including a diuretic, and affects roughly 10% of individuals with hypertension, although estimates vary by criteria and adherence assessment (4). Excess aldosterone, even below diagnostic thresholds for primary aldosteronism, contributes importantly to treatment resistance (11, 12, 13, 14, 15).

Directly reducing aldosterone biosynthesis via selective CYP11B2 inhibition offers a complementary strategy that may reduce the risk of hyperkalemia associated with MR blockade and addresses the hormonal source of treatment resistance. Aldosterone synthase (CYP11B2) catalyzes the terminal steps of aldosterone production, whereas 11β-hydroxylase (CYP11B1) generates cortisol. Selectivity has been challenging, owing to the high homology between CYP11B2 and CYP11B1. However, recent advances in structural biology have enabled structure-guided design, yielding candidates with improved selectivity. These aldosterone synthase inhibitors (ASIs) aim to suppress aldosterone while minimizing interference with cortisol synthesis and with upstream steroidogenic precursors. The selective inhibition of aldosterone synthase (CYP11B2) remains technically challenging because CYP11B2 and the cortisol synthase CYP11B1 share ∼93% amino acid identity and overlapping substrate recognition (12). Earlier ASI programs, most notably LCI699 (osilodrostat), achieved blood pressure reductions in clinical studies but were limited by dose-dependent suppression of ACTH-stimulated cortisol. Aromatase-derived scaffolds, such as fadrozole/FAD286, lacked sufficient CYP11B2 selectivity and inhibited CYP11B1 in human adrenal models, underscoring the need for greater CYP11B2 selectivity. Recent structural work on human CYP11B2 has highlighted active-site features (e.g. Trp-116, Arg-120, and Phe-487) that can be exploited to design selective inhibitors. What remains scarce, however, are head-to-head assessments of selectivity in primary human adrenocortical cells, the most physiologically relevant *in vitro* system for steroidogenesis.

Clinically, baxdrostat, a selective ASI with >100-fold *in vitro* selectivity for CYP11B2 over CYP11B1, has demonstrated efficacy in hard-to-control hypertension. In the phase 2 BrigHTN trial, least-squares mean changes in systolic blood pressure at 12 weeks were −20.3 mmHg (2 mg), −17.5 mmHg (1 mg), and −12.1 mmHg (0.5 mg) versus −9.4 mmHg with placebo; the placebo-adjusted differences were −11.0 mmHg (2 mg) and −8.1 mmHg (1 mg), with favorable tolerability and no adrenal insufficiency reported (16).

In the phase 3 BaxHTN trial (on top of standard care), placebo-adjusted reductions in seated systolic blood pressure at 12 weeks were −9.8 mmHg (2 mg) and −8.7 mmHg (1 mg); baxdrostat was generally well tolerated with low rates of confirmed hyperkalemia (∼1.1% per active arm) and no cases of adrenal insufficiency (17).

Unlike transformed cell lines or rodent models, primary human adrenocortical cultures retain the donor-specific expression of steroidogenic enzymes, hormone responsiveness, and pathway regulation. This approach enables evaluation of drug effects on the full spectrum of adrenal steroid outputs, including mineralocorticoids, glucocorticoids, and androgens, under near-physiological conditions, bridging the gap between molecular binding assays and clinical outcomes.

We hypothesize that selective CYP11B2 inhibition suppresses aldosterone with minimal impact on cortisol synthesis or upstream precursor accumulation, whereas nonselective CYP11B1/B2 inhibition produces broader steroidogenic disruption. To test this hypothesis, we systematically compare three CYP11B inhibitors with differing CYP11B2/CYP11B1 selectivity profiles in primary human adrenal cell cultures derived from aldosterone-producing adenomas (APAs), cortisol-producing tumors (CPTs), and histologically normal adrenal cortex (NAG, resected adjacent to pheochromocytomas). We quantify dose–response behavior (IC_50_ as geometric mean (GM) with 95% CIs), assess steroid panels by LC–MS/MS to capture on-target and off-target pathway effects, and perform molecular docking to rationalize enzyme–ligand interactions at CYP11B2 versus CYP11B1. This integrated platform links molecular selectivity to functional steroid profiles, informing therapeutic development and clinical translation of selective aldosterone suppression.

## Materials and methods

### Participants and specimen classification

Adrenocortical tissues were collected from 23 patients who underwent adrenalectomy (August 2022–September 2024): APAs (*n* = 10), CPTs (adenomas, *n* = 9; carcinoma, *n* = 1), adrenal cortical tissue adjacent to APAs or CPTs, and histologically NAG (*n* = 3) resected as nontumorous cortex adjacent to pheochromocytomas. Diagnostic assignment followed standard criteria: APA, biochemical primary aldosteronism with CYP11B2-positive adenoma; CPT, autonomous cortisol secretion with CYP11B1-positive tumor; and NAG, normal cortical architecture with expected enzyme expression.

### Exclusion criteria

Patients with prior exposure to steroidogenesis-modifying agents (e.g. mitotane, ketoconazole, metyrapone, and systemic glucocorticoids) were excluded.

### Human adrenal tissues and primary cell culture

Primary human adrenal cells were isolated from APAs, CPTs, adrenal cortical tissue adjacent to APAs or CPTs, and histologically NAG (resected adjacent to pheochromocytomas) as described (18, 19). Cells were seeded on Matrigel-coated plates at ∼0.5–1 × 10^5^ cells/well in DMEM/F12 supplemented with 5% fetal bovine serum and 1% penicillin/streptomycin, allowed to attach for 72–96 h, and then exposed to test conditions in fresh medium. Vehicle controls contained ≤0.1% dimethyl sulfoxide (DMSO). Detailed procedures are described in the Supplementary Methods (see section on Supplementary materials given at the end of the article).

### Compounds and cell treatment

Baxdrostat (a selective CYP11B2 inhibitor), osilodrostat (primarily a CYP11B1 inhibitor), and metyrapone (a broad 11β-hydroxylase inhibitor) were dissolved in DMSO and subsequently diluted in growth medium (final DMSO ≤ 0.1%). Unless otherwise specified, cells were treated with these compounds for 72 h at concentrations ranging from 0 to 10 μM with ACTH. ACTH concentrations (10 nM) were selected based on preliminary dose–response data for aldosterone and cortisol production to provide robust stimulation without reaching saturation (19).

### Primary cultures with protein-based normalization and RT-qPCR

To facilitate protein normalization and reverse-transcription quantitative PCR (RT-qPCR) analyses, additional primary cell cultures were established from four newly acquired adrenal tumor samples (two APA and two CPT cases). All samples were collected at the same institution and surgical department and in accordance with the same IRB protocol as the original cohort. Identical primary culture procedures were employed throughout. Cells were seeded into 12-well plates at a target density of 2 × 10^5^ cells/well. Following a 72-h conditioning period, the medium was replaced with fresh medium containing ACTH (10 nM) and either baxdrostat, osilodrostat, or vehicle control and incubation continued for an additional 72 h before conditioned media were collected.

After medium collection, cells were processed using TRIzol reagent (Invitrogen, USA) according to the manufacturer’s instructions. Total cellular protein was isolated, solubilized in 1% sodium dodecyl sulfate (SDS), and quantified using the Pierce BCA Protein Assay Kit – Reducing Agent Compatible (Thermo Fisher Scientific, USA). Aldosterone and cortisol concentrations in the conditioned media were normalized to total cellular protein content and compared with values normalized to the intended seeding cell number.

### RNA isolation, cDNA synthesis, and RT-qPCR

Total RNA was isolated from the TRIzol aqueous phase in accordance with the manufacturer’s instructions and subsequently reverse-transcribed into cDNA. qPCR assays for CYP11B1, CYP11B2, and CYP17A1 were conducted using SYBR green chemistry on a StepOnePlus Real-Time PCR System (Applied Biosystems, Foster City, USA). Transcript levels were normalized to the ribosomal protein L32 (*RPL32*; L32) as an internal reference gene, and the relative expression was determined using the 2^−ΔΔCt^ method (20), with the mean ΔCt of vehicle-treated wells within each case serving as the calibrator.

### Steroid quantification by LC–MS/MS

At 72 h, supernatants were collected for LC–MS/MS quantification of steroid panel members (aldosterone, cortisol, corticosterone, 11-deoxycorticosterone [11-DOC], 11-deoxycortisol [11-DOF], 17-hydroxyprogesterone [17-OHP], progesterone, dehydroepiandrosterone (DHEA), and related precursors) using stable-isotope-labeled internal standards and external calibration. Results are reported as percent change vs vehicle unless noted otherwise.

### Immunohistochemistry

Immunohistochemistry (IHC) was performed on 4 μm formalin-fixed paraffin-embedded (FFPE) sections using the Ventana Discovery Ultra platform (Ventana Medical Systems, USA) with ChromoMap DAB detection. Primary antibodies included anti-CYP11B1 (1:400, ab70198, Abcam, UK), anti-CYP11B2 (1:800), anti-CYP17A1 (1:1,000, ab134938, Abcam), and anti-CD56 (clone 123C3, M730429-2, Agilent Technologies, USA).

### Molecular docking

Receptor models for human CYP11B2 and CYP11B1 were prepared from published crystal structures (waters/ions treated per standard protocols; heme parameters retained as appropriate). Docking was performed with AutoDock Vina (v1.2.5) using grid boxes centered on the catalytic heme; results are reported as Vina scores (kcal/mol), and top-ranked poses were inspected in UCSF ChimeraX for catalytic geometry and steric compatibility. Full settings and PDB identifiers are listed in Supplementary Methods (12, 21, 22).

### Statistical analysis

Dose–response curves were fitted to a three-parameter logistic (3PL) model with top fixed at 100% (responses normalized to vehicle) and bottom constrained ≥ 0; the Hill slope was fixed at −1.0, and IC_50_ was estimated from the fitted curves. Span was defined as the dynamic range of the fitted curve (top − bottom; with top fixed at 100% in 3PL fits). IC_50_ values are summarized as GMs with 95% CIs computed using asymmetric profile likelihood. IC_50_ comparisons used a mixed-effects model of log-transformed IC_50_ values (donor as a random effect), with Tukey’s adjustment for multiple comparisons. Span (top − bottom) and other multi-group endpoints were analyzed using the Kruskal–Wallis test with Dunn’s post hoc test. Curves not achieving ≥50% inhibition within 0–10 μM at 72 h were labeled NE (not estimable) and excluded from ratio-based summaries. Two-sided tests were used with *α* = 0.05. Analyses were performed in GraphPad Prism (v10.4) (GraphPad Software, USA) and R (v4.01). For the additional RT-qPCR analyses, group differences in ΔCt were evaluated using the Kruskal–Wallis test with Dunn’s post hoc test. To objectively determine whether mRNA expression levels remained within a pre-specified descriptive range relative to the vehicle control, equivalence testing was conducted using the two one-sided tests (TOST) procedure (23) with an equivalence bound of ±1 ΔCt cycle (equivalent to ±2 FC). This descriptive range is frequently used in qPCR practice; however, it is not defined by any formal guidelines. Formal equivalence at this bound was concluded when the 90% CI of ΔΔCt lie entirely within ±1 cycle (*P*_equiv < 0.05). Spearman correlations were used to compare hormone values normalized to protein with those normalized to nominal cell number.

## Results

### Patient and tumor characteristics

**Figure 1:**
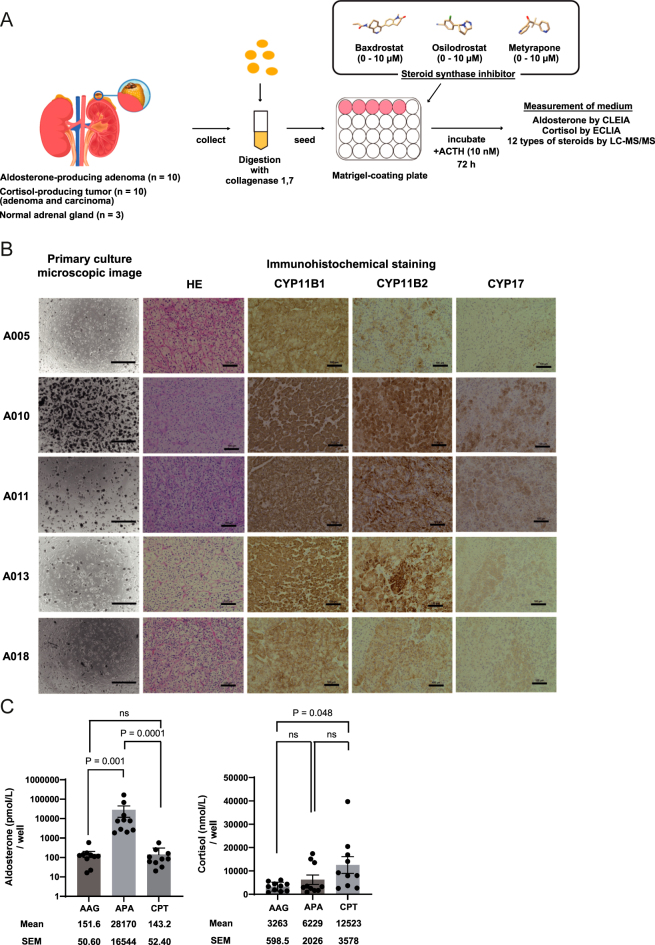
Study workflow and baseline steroid secretion in primary adrenal cultures. (A) Overview of tissue sampling, dissociation, and culture assay. (B) Representative images of primary cultured cells and hematoxylin–eosin staining, as well as immunohistochemical staining, in adrenal tissue from representative APA patients. (C) Baseline steroid secretion at 72 h under vehicle (no inhibitor) across aldosterone-producing adenoma (APA), cortisol-producing tumor (CPT), and adjacent/normal adrenal gland (AAG). In this figure, AAG includes histologically normal adrenal cortex resected adjacent to pheochromocytoma, as well as the adrenal region adjacent to APA or CPT. Data are mean ± SEM across donors; *n* shown in panels. Statistics: Kruskal–Wallis with Dunn’s post hoc test (two-sided *α* = 0.05). APA, aldosterone-producing adenoma; CPT, cortisol-producing tumor; AAG, adjacent/normal adrenal gland; HE, hematoxylin and eosin staining.

Primary adrenocortical cultures were established from 23 surgical specimens (Fig. 1A): ten APAs, ten CPTs (nine adenomas and one carcinoma), and three histologically normal adrenal cortices (NAG) adjacent to pheochromocytomas (Table 1). These three tissue types were used to compare the effects of baxdrostat and osilodrostat on steroidogenesis.

**Table 1:** Clinical and pathological characteristics of patients.

Patient no.	Sex	Clinical diagnosis	Blood pressure (mmHg)	Age at surgery (years)	PRA (ng/mL/h)	PAC (pmol/L)	ACTH (pmol/L)	F (nmol/L)	Serum potassium level (mmol/L)	F (nmol/L) after 1 mg dexamethasone suppression test	Tumor side	Size of lesion (cm)	Pathological diagnosis	Weiss score
**Aldosterone-producing adenoma**														
A005	Female	PA	139/91	38	0.8	507.6	3.6	251.1	2.3	22.1	Left	1.6 × 1.2 × 1.5	Adrenocortical adenoma	1
A009	Female	PA	120/59	42	<0.2	1,142.9	5.7	289.7	2.5	27.6	Right	1.1 × 1.1 × 1.3	Adrenocortical adenoma	0
A010	Female	PA SCS	141/82	70	0.5	721.2	2.0	303.5	3.1	69.0	Right	1.8 × 2.3 × 2.3	Adrenocortical adenoma	0
A011	Male	PA	142/92	61	0.6	857.2	4.7	281.4	2.4	49.7	Left	1.9 × 2.1 × 1.9	Adrenocortical adenoma	0
A013	Female	PA	146/96	47	0.3	532.6	5.1	182.1	2.8	19.3	Right	1.6 × 2.0 × 2.1	Adrenocortical adenoma	Not reported
A018	Female	PA SCS	128/63	54	0.7	804.5	0.8	140.7	2.6	126.9	Left	2.3 × 2.6 × 2.4	Adrenocortical adenoma	0
R001	Female	PA	150/80	50	<0.2	968.1	3.8	138.0	3.2	ND	Right	1.5 × 1.2 × 1.0	Adrenocortical adenoma	0
R002	Male	PA	224/142	48	<0.2	435.5	7.7	344.9	3.3	24.8	Left	1.6 × 1.2 × 0.7	Adrenocortical adenoma	0
R003	Female	PA	183/96	73	<0.2	865.5	16	331.1	3.2	41.4	Right	1.5 × 1.3 × 1.7	Adrenocortical adenoma	0
R004	Male	PA	152/90	66	0.2	471.6	11.8	303.5	2.5	16.6	Right	1.6 × 1.4 × 1.6	Adrenocortical adenoma	1
**Cortisol-producing tumor**														
C003	Female	SCS	189/93	57	1.0	235.5	1.1	562.8	3.7	278.7	Right	3.0 × 2.8 × 2.9	Adrenocortical adenoma	0
C004	Female	SCS	116/68	41	0.4	108.2	<0.3	229.0	3.9	278.7	Left	2.4 × 1.5 × 2.0	Adrenocortical adenoma	0
C005	Female	OCS	191/75	71	1.1	126.5	<0.3	306.3	3.8	306.2	Right	2.9 × 2.7 × 3.5	Adrenocortical adenoma	0
C013	Female	OCS	142/88	49	0.5	81.8	<0.3	223.5	3.7	218.0	Right	1.7 × 2.6 × 2.4	Adrenocortical adenoma	0
C015	Female	SCS	130/80	73	0.4	<11.1	0.4	295.2	4.3	205.5	Right	1.4 × 2.2 × 1.9	Adrenocortical adenoma	0
C016	Female	SCS	142/75	47	3.0	132.6	2.4	173.8	4.0	216.0	Right	2.7 × 2.0 × 2.4	Adrenocortical adenoma	0
C018	Female	ACC OCS	164/93	65	0.4	53.5	<0.3	278.7	3.8	241.4	Left	4.3 × 5.5 × 4.8	Adrenocortical carcinoma	8
C019	Male	SCS	192/131	52	0.5	154.8	<0.3	118.6	3.9	135.2	Left	2.2 × 2.4 × 2.1	Adrenocortical adenoma	0
C020	Female	SCS	89/72	36	2.5	196.7	<0.3	171.1	4.4	143.5	Left	1.8 × 2.4 × 1.7	Adrenocortical adenoma	1
C021	Female	SCS	107/63	54	ND	41.1	<0.3	212.4	3.9	193.1	Right	2.1 × 3.6 × 3.0	Adrenocortical adenoma	0
**Normal adrenal gland**														
N001	Female	Pheo	128/89	32	2.3	218.0	10.2	353.2	4.0	ND	Right	3.0 × 2.5 × 2.2	Pheochromocytoma	
N003	Female	Pheo	114/64	71	0.3	139.5	4.7	400.1	3.9	ND	Left	4.0 × 2.0 × 1.0	Pheochromocytoma	
N004	Female	Pheo	96/59	27	13.4	240.0	3.9	162.8	4.0	ND	Right	4.0 × 2.5 × 1.0	Pheochromocytoma	

Tissue diagnosis was based on the pathology report. Blood pressure is indicated as systolic/diastolic. Plasma ACTH, PAC, and F values were converted to SI units using the following factors: ACTH (1 pg/mL = 0.2202 pmol/L), PAC (1 pg/mL = 2.774 pmol/L), and F (1 μg/dL = 27.59 nmol/L). PRA, plasma renin activity; PAC, plasma aldosterone concentration; ACTH, adrenocorticotropic hormone; F, cortisol; PA, primary aldosteronism; SCS, subclinical Cushing’s syndrome; OCS, overt Cushing’s syndrome; ACC, adrenocortical carcinoma; Pheo, pheochromocytoma; ND, no data.

The immunohistochemistry of matched FFPE tissues confirmed expected enzyme expression patterns (Fig. 1B, Supplementary Figs 1, 2, 3): APAs were CYP11B2-positive; CPTs expressed CYP11B1 and CYP17A1 but lacked CYP11B2; and NAG expressed CYP11B1 and CYP17A1.

### Characterization of primary adrenocortical cell cultures

Culture identity, phenotype, and basal steroid secretion were confirmed by immunofluorescence (IF), flow cytometry, and CD56 as an adrenocortical marker. IF showed CD56^+^ cells across APA, CPT, and NAG cultures, with stronger signals in APA (Supplementary Figs 4 and 5). Flow cytometry quantified viable CD56^+^ cells ranging from 7.3 to 64.8% of adherent cells (Supplementary Figs 6, 7, 8, Supplementary Table 1).

At 72 h (Fig. 1C), aldosterone was higher in APA (28,170 ± 16,544 pmol/L) vs CPT (143.2 ± 52.40 pmol/L; *P* < 0.0001) and adjacent/normal adrenal gland (AAG) (151.6 ± 50.60 pmol/L; *P* < 0.001). Cortisol was the highest in CPT (12,523 ± 3,578 nmol/L) vs AAG (3,263 ± 598.5 nmol/L; *P* = 0.048) and not significant vs APA (6,229 ± 2,026 nmol/L). These findings indicate that the primary cultures preserve tissue-specific steroidogenic phenotypes (aldosterone-enriched APA; cortisol-enriched CPT).

### Baxdrostat and osilodrostat potently inhibit aldosterone synthesis in APA cells

**Figure 2:**
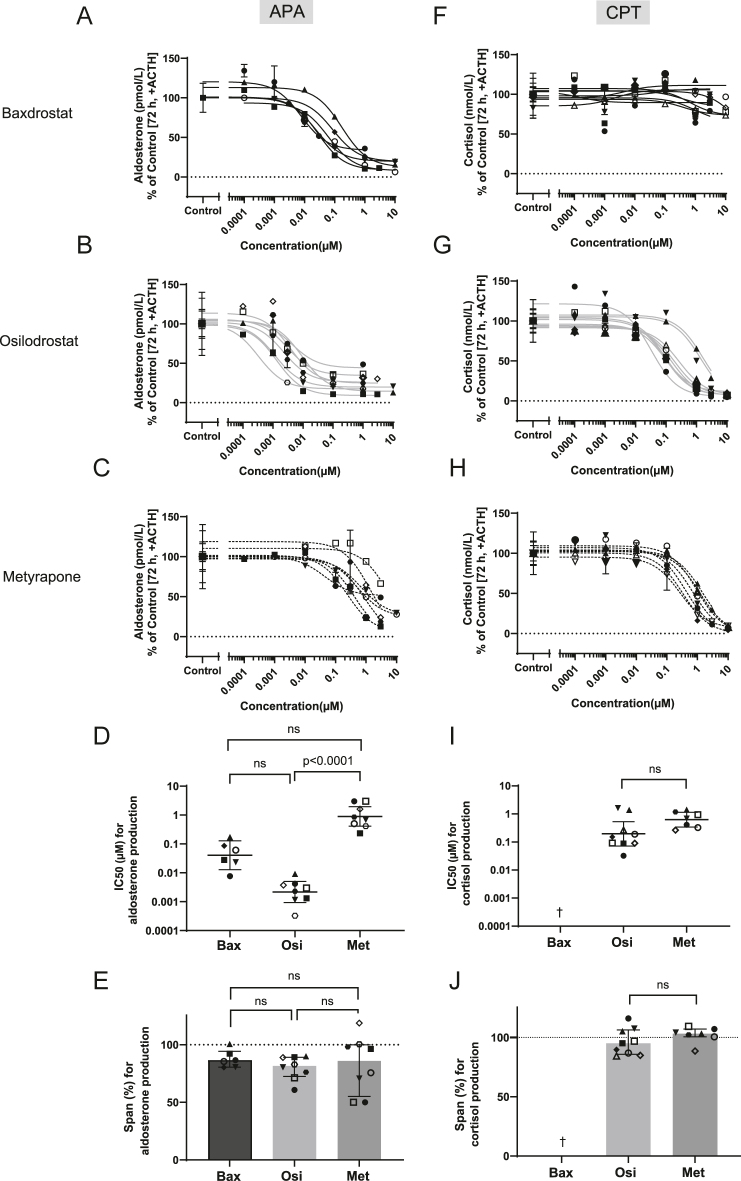
Dose–response to aldosterone synthase inhibitors across APA and CPT primary cultures. (A, B, C) Baxdrostat (A), osilodrostat (B), and metyrapone (C) dose–response curves for aldosterone in APA after 72 h with ACTH 10 nM. (D and E) Geometric mean (GM) IC_50_ (D) and maximum inhibition (span) (E) for aldosterone in APA. (F, G, H) Baxdrostat (F), osilodrostat (G), and metyrapone (H) dose–response curves for cortisol in CPT after 72 h with ACTH 10 nM. (I and J) GM IC_50_ (I) and spans (J) for cortisol in CPT. ^†^Baxdrostat did not reach ≥50% cortisol inhibition within 0–10 μM at 72 h; IC_50_ and span were NE. Data are mean ± SEM across donors; *n* shown in panels. Model/normalization: curves were fitted with a three-parameter logistic (3PL) model (top fixed at 100%; bottom ≥ 0); responses are % vehicle (72 h). Span = top – bottom, expressed as %. Statistics: IC_50_ comparisons used a mixed-effects model on log-transformed IC_50_ (donor as a random effect) with Tukey’s adjustment for multiple comparisons. Multi-group endpoints, including span, were analyzed using the Kruskal–Wallis test with Dunn’s post hoc test (two-sided *α* = 0.05). Significance: *P* < 0.05; *P* < 0.01; *P* < 0.001; ns, not significant. Units/axes: concentration (μM); response (% of vehicle at 72 h). ACTH, adrenocorticotropic hormone; APA, aldosterone-producing adenoma; CPT, cortisol-producing tumor; IC_50_, half-maximal inhibitory concentration; NE, not estimable. ^†^IC_50_/span not estimable (≥50% inhibition not reached).

Across APA, all inhibitors produced concentration-dependent suppression of aldosterone ((Fig. 2A, B, C, D, E, Table 2, Supplementary Table 2; GM IC_50_ (μM): baxdrostat 0.0407 (95% CI: 0.0129–0.129; *n* = 6), osilodrostat 0.00217 (0.000933–0.00504; *n* = 8), and metyrapone 0.894 (0.410–1.950; *n* = 8; Supplementary Table 3)). The curves used the three-parameter logistic model (bottom ≥ 0); responses not reaching ≥50% inhibition within 0–10 μM/72 h were NE.

**Table 2:** Efficacy of baxdrostat, osilodrostat, and metyrapone for aldosterone and cortisol production by human primary adrenocortical cultures.

Patient no.	Symbol	IC_50_ value			Span		
		Baxdrostat	Osilodrostat	Metyrapone	Baxdrostat	Osilodrostat	Metyrapone
**Aldosterone-producing adenoma (for aldosterone)**							
A005	●	0.0077 (0.0032–0.0194)	0.0042 (NE–12.840)	>3.0000	86.6	60.8	<50.0
A009	■	0.0280 (0.0126–0.0622)	0.0013 (0.0008–0.0022)^a^	0.2337 (0.1270–0.4280)^b^^,^^d^	92.2	89.2	96.4
A010	▲	0.1727 (0.0618–0.5109)	0.0091 (0.0043–0.0196)^b^	NT	100.6	89.9	NT
A011	▼	0.0234 (0.0175–0.0315)	0.0012 (0.0008–0.0016)^a^	0.6845 (0.1212–2.7650)^b^^,^^d^	80.6	80.5	70.8
A013	♦	0.0860 (0.0726–0.1016)	NT	NT	80.4	NT	NT
A018	〇	0.0611 (0.0150–0.2100)	NT	0.5021 (0.3644–0.6635)	85.4	NT	75.7
R001	□	NT	0.0030 (0.0012–0.0076)	>3.0000^e^	NT	71.2	<50.0
R002	⬣	NT	0.0023 (0.0004–0.0133)	0.8525 (NE–12.980)^f^	NT	76.2	98.2
R003	♢	NT	0.0037 (0.0006–0.0358)	1.3340 (NE–NE)^f^	NT	89	118.8
R004	⬡	NT	0.0003 (NE–0.0022)	0.4103 (NE–5.4750)^f^	NT	82.8	100.5
**Cortisol-producing tumor (for cortisol)**							
C003	●	NC	0.0325 (0.0050–0.1901)	0.4125 (0.1530–0.7024)^f^	NC	116.1	107.1
C004	■	NC	0.0890 (0.0645–0.1226)	NT	NC	95.2	NT
C005	▲	NC	1.3910 (0.5980–2.0940)	1.4200 (0.6668–2.0730)	NC	105.3	103.3
C013	▼	NC	1.6400 (0.0550–3.8000)	0.6923 (0.0762–1.6280)	NC	107.5	104.2
C015	♦	NC	0.1522 (0.0180–0.9174)	NT	NC	89.7	NT
C016	〇	NC	0.1892 (0.0542–0.5800)	0.3275 (0.0837–0.8583)	NC	86.9	100.6
C018	□	NC	0.0919 (0.0237–0.3056)	0.9364 (0.2180–2.5110)^f^	NC	97	109.4
C019	△	NC	0.2676 (0.0680–0.9194)	NT	NC	84.4	NT
C020	⬣	NT	NT	1.1780 (0.6199–1.6700)	NT	NT	102.2
C021	♢	NC	0.0906 (0.0353–0.2219)	0.2659 (0.1076–0.6475)	NC	84.9	88.6
**Normal adrenal gland (for cortisol)**							
N003	■	>10.000	0.1889 (0.1066–0.3398)^c^	0.2309 (0.1159–0.4058)^c^	<50.0	91.7	102.7
N004	▲	>10.000	NT	NT	<50.0	NT	NT

IC_50_ values are presented in micromolar units and reported with 95% confidence intervals (CIs) using a three-parameter logistic (3PL) model with bottom > 0 constraints. Span is a derived quantity (top − bottom) and is reported without a CI; its uncertainty should be inferred from the CIs of top and bottom (Supplementary Table 2). The symbols are used in Fig. 2. Patient N001 is not displayed because only the baseline value was measured, and no drug was added. For cases where the maximum concentration did not achieve 50% inhibition, IC_50_ was defined as > maximum concentration, and span was defined as <50.0%. NE, not estimable; NT, not tested; NC, not calculable.

^a^ *P* < 0.0001 compared with the IC_50_ of baxdrostat.

^b^ *P* < 0.001 compared with the IC_50_ of baxdrostat.

^c^ *P* < 0.01 compared with the IC_50_ of baxdrostat.

^d^ *P* < 0.0001 compared with the IC_50_ of osilodrostat.

^e^ *P* < 0.01 compared with the IC_50_ of osilodrostat.

^f^ *P* < 0.05 compared with the IC_50_ of osilodrostat.

Mixed-effects analysis of log (IC_50_) indicated that osilodrostat was ∼11-fold more potent than baxdrostat (GMR: 11.1; 95% CI: 0.91–134.6; *P* = 0.06), baxdrostat was ∼30-fold more potent than metyrapone (GMR: 0.0337; 0.000759–1.489; *P* = 0.07), and osilodrostat > metyrapone (GMR: 0.00182; 0.000845–0.00391; *P* < 0.001; Fig. 2D, Supplementary Table 4). Within-donor pairs (*n* = 4–7) were concordant.

To contextualize potency, baxdrostat showed a GM IC_50_ for aldosterone of 0.041 μM (APA) versus 0.0022 μM for osilodrostat and 0.894 μM for metyrapone, establishing a clear rank order of aldosterone suppression (osilodrostat < baxdrostat << metyrapone). Selectivity profiles diverged accordingly: baxdrostat IC_50_ for cortisol was orders of magnitude higher than for aldosterone, whereas osilodrostat inhibited both pathways within the submicromolar range. Across donors, between-drug differences in aldosterone IC_50_ were significant in the pre-specified model with donor as a random effect and in multiplicity-controlled post hoc comparisons, supporting a robust selectivity advantage for baxdrostat.

### Baxdrostat preserves cortisol synthesis in CPT and normal adrenal cells

To assess pathway-wide effects, we profiled 12 steroids by LC–MS/MS in supernatants from wells treated at concentrations approximating IC_50_ or maximal inhibition (Supplementary Figs 10, 11, 12, Supplementary Tables 7, 8, 9).

**Figure 3:**
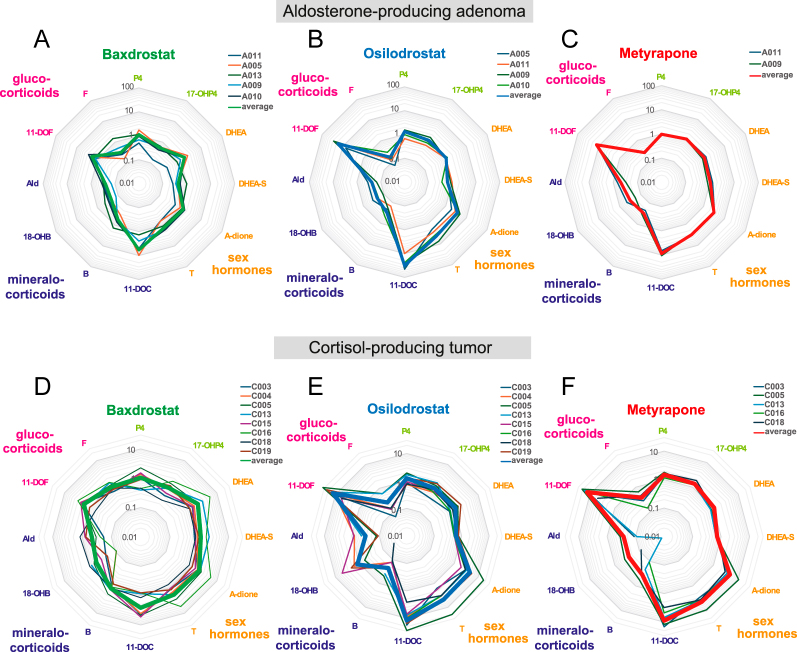
Steroidogenic profiling of aldosterone synthase inhibitors in APA and CPA primary cultures. (A, B, C) Radar plots showing fold change (drug/vehicle) for each steroid in APA cultures after 72 h treatment with 1 μM baxdrostat (A), osilodrostat (B), or metyrapone (C) under ACTH 10 nM. (D, E, F) The same for CPT cultures. 1 μM dose represents a suprapharmacological concentration selected to accentuate between-drug differences. Data are median (IQR). APA: *n* = 5 (baxdrostat), *n* = 4 (osilodrostat), *n* = 2 (metyrapone). CPT: *n* = 8 (baxdrostat), *n* = 8 (osilodrostat), *n* = 5 (metyrapone). ACTH, adrenocorticotropic hormone; Bax, baxdrostat; Osi, osilodrostat; Met, metyrapone; Ald, aldosterone; F, cortisol; B, corticosterone; 11-DOC, 11-deoxycorticosterone; 11-DOF, 11-deoxycortisol; 18-OHB, 18-hydroxycorticosterone; 17-OHP4, 17-hydroxyprogesterone; A-dione, androstenedione; DHEA, dehydroepiandrosterone; DHEA-S, dehydroepiandrosterone sulfate; T, testosterone; P4, progesterone.

In APA, baxdrostat selectively reduced aldosterone (−72.9 ± 3.6%) and 18-hydroxycorticosterone (−73.2 ± 5.0%), with 11-DOC accumulation (+376.4 ± 146.2%); cortisol and 17-OHP decreased (−46.0 ± 24.5%; −52.1 ± 9.2%). By contrast, osilodrostat caused greater 11-DOC (+2,539.2 ± 720.7%; *P* < 0.05 vs baxdrostat), increased 11-DOF (+1,571.5 ± 421.5%; *P* < 0.05), and higher androgens (androstenedione: +354.6 ± 147.8%; *P* < 0.05), consistent with dual CYP11B2/CYP11B1 inhibition (Fig. 3A, B, C, Supplementary Fig. 10).

Precursor patterns aligned with pharmacology. In APA cultures, 11-DOC increased ∼3.8-fold with baxdrostat at 1 μM but ∼25-fold with osilodrostat; 11-DOF and corticosterone showed similar rank-order changes, consistent with greater CYP11B1 blockade by osilodrostat. By contrast, baxdrostat minimized precursor accumulation while suppressing aldosterone, indicating preserved 11β-hydroxylase activity. This profile is associated with a lower risk of MR-mediated sodium retention from precursor buildup compared with nonselective inhibition.

In CPT, aldosterone was low at baseline. Baxdrostat did not reach ≥50% cortisol inhibition within 0–10 μM (IC_50_ NE), indicating CYP11B2 selectivity (Fig. 2F, G, H, I, J, Supplementary Fig. 11). Osilodrostat and metyrapone suppressed cortisol (GM IC_50_: 0.196 and 0.629 μM, respectively; Supplementary Table 5). In two CPT cultures with overt Cushing’s (C005, C013), osilodrostat curves were right-shifted, indicating lower potency (Fig. 2G, Table 2).

In NAG, patterns were consistent (Supplementary Figs 9 and 12, Supplementary Table 9) (baxdrostat: cortisol −20.7 ± 11.2%, corticosterone −16.9 ± 7.7%, IC_50_ NE; osilodrostat: cortisol −90.3 ± 0.9%, corticosterone −89.9 ± 1.8%, with 11-DOC +696.3 ± 30.3% and 11-DOF +1,916.6 ± 812.2%).

In histologically NAG, the preservation of cortisol synthesis was notable: baxdrostat maintained >75% of baseline cortisol at 10 μM, whereas osilodrostat reduced cortisol to ∼10% of baseline at the same concentration, accompanied by marked accumulation of 11-DOF/11-DOC. Aldosterone was suppressed by both drugs, but upstream perturbations were substantially smaller with baxdrostat. These *ex vivo* differences across NAG donors underscore the clinical relevance of CYP11B2 selectivity for limiting iatrogenic adrenal insufficiency while targeting aldosterone.

### Validating protein-based normalization in additional primary cultures

In supplementary validation studies involving four newly acquired adrenal tumor samples (two APA and two CPT cases) at the same institution and under the original IRB protocol, notable variability was observed in total cellular protein across wells (mean ± SD = 50.2 ± 11.2 μg/well; range: 29.3–75.0 μg/well; coefficient of variation = 22%), suggesting that initial seeding density did not fully account for cell mass following culture. Despite this, hormone levels normalized to nominal cell number exhibited a strong correlation with those normalized to total protein for both aldosterone (Spearman *r* = 0.9912 on a log_10_ scale, *n* = 18, *P* < 0.0001) and cortisol (*r* = 0.8142 on a log10 scale, *n* = 18, *P* < 0.0001). Moreover, the direction and magnitude of the primary drug-induced effects were largely maintained following protein normalization (Supplementary Figs 13 and 14). These findings support applying protein-normalized analysis to validate the sensitivity of the original observations, rather than conducting a retrospective reanalysis of the entire cohort.

### Molecular docking predicts a structural basis for baxdrostat selectivity

**Figure 4:**
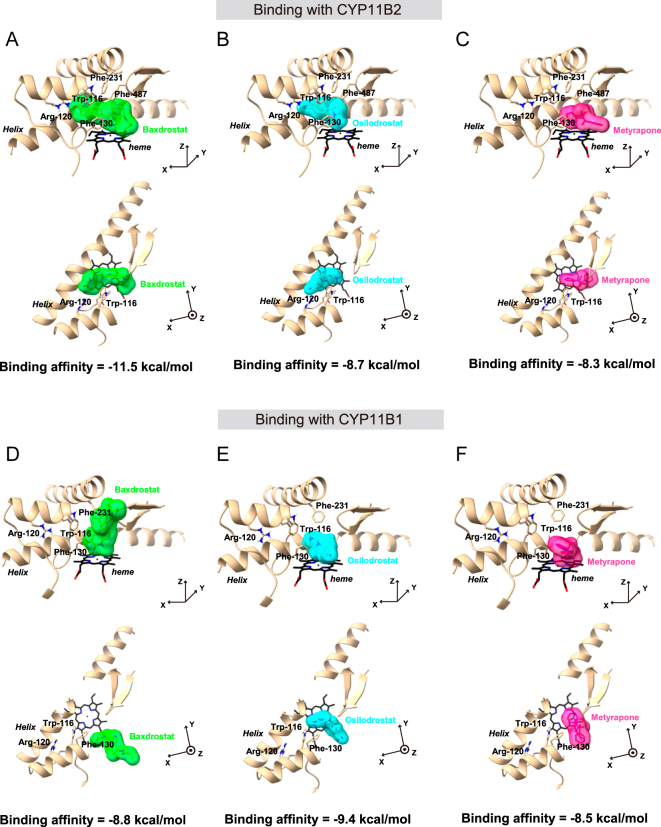
Molecular docking of aldosterone synthase inhibitors to CYP11B2 and CYP11B1. (A, B, C) Predicted binding poses of baxdrostat (A), osilodrostat (B), and metyrapone (C) in the CYP11B2 (aldosterone synthase) active site. (D, E, F) Corresponding poses of the same inhibitors in CYP11B1 (11β-hydroxylase). Representative interacting residues are annotated to illustrate pocket differences between CYP11B2 and CYP11B1. Schematic interaction diagrams accompany the 3D poses. Qualitative concordance: selective CYP11B2 engagement by baxdrostat and comparatively broader CYP11B1 engagement by osilodrostat/metyrapone align with primary-culture bioactivity (see Figs 2 and 3). CYP11B2, aldosterone synthase; CYP11B1, 11β-hydroxylase.

Docking simulation (Fig. 4) supported these results. In CYP11B2, baxdrostat adopted a deep pose with heme Fe–N coordination (ΔG −11.5 kcal/mol); osilodrostat/metyrapone scored −8.7/−8.3. In CYP11B1, baxdrostat bound peripherally (−8.8), whereas osilodrostat/metyrapone coordinated heme (−9.4/−8.5). The rank order matched IC_50_ and steroid profiles.

Selective CYP11B2 inhibition suppressed aldosterone with limited perturbation of glucocorticoid/mineralocorticoid pathways, whereas less selective 11β-hydroxylase inhibition caused precursor accumulation, glucocorticoid depletion, and androgen shunting. Concordant evidence from IC_50_, within-donor pairs, LC–MS/MS panels, and docking explains the distinct pharmacologic profiles.

## Discussion

Across primary human adrenocortical cells (APA/CPT/NAG), convergent evidence from functional assays, LC–MS/MS profiling, and molecular docking demonstrates that baxdrostat selectively inhibits CYP11B2. By contrast, osilodrostat inhibits both CYP11B2 and CYP11B1, extending observations from recombinant/immortalized systems to a physiologically relevant *ex vivo* model (13, 24). Importantly, human exposures overlap our APA potency range: reported C_max ∼0.028–0.110 μM (0.5–2 mg); GM IC_50_ ∼0.04 μM for aldosterone, with dose-proportional pharmacokinetics (T_max = 4 h; t½ ∼26–31 h), parameters consistent with clinically relevant aldosterone suppression without excessive enzyme blockade (25).

In NAG cultures, baxdrostat did not achieve ≥50% cortisol inhibition at 0–10 μM, consistent with clinical data showing no adrenal insufficiency signal (16, 17).

The translational concordance is supported by phase 2/3 data. In BrigHTN (phase 2; *n* = 248), 12-week least-squares mean changes in seated systolic BP were −20.3 (2 mg), −17.5 (1 mg), and −12.1 mmHg (0.5 mg) vs −9.4 mmHg with placebo; placebo-adjusted differences were −11.0 mmHg (2 mg) and −8.1 mmHg (1 mg) (16).

In BaxHTN (phase 3; *n* = 796) on top of standard care, placebo-adjusted reductions at 12 weeks were −9.8 (2 mg) and −8.7 mmHg (1 mg); serum potassium > 6.0 mmol/L occurred in ∼2–3% in active arms, with no adrenal insufficiency reported. These effects were consistent across uncontrolled and resistant subgroups (17).

Together with our IC_50_ data, therapeutic unbound concentrations are predicted to achieve substantial aldosterone suppression while preserving >90% cortisol, supporting model-to-clinic concordance.

Steroidogenic signatures differentiated the agents. In APA, baxdrostat reduced aldosterone and 18-hydroxycorticosterone with moderate 11-DOC accumulation (∼376%), mirroring the transient 11-DOC elevations observed with selective CYP11B2 inhibition (26). Docking rationalized selectivity and strong heme coordination in CYP11B2 (ΔG −11.5 kcal/mol) with weaker, distal engagement in CYP11B1 (ΔG −8.8 kcal/mol), consistent with preserved 11β-hydroxylase. By contrast, osilodrostat produced ∼seven-fold greater 11-DOC buildup (∼2,539%) and marked increases in 11-DOF across APA/CPT/NAG, indicating dual blockade. Although 11-DOC binds MR with ∼50% of aldosterone’s affinity, its *in vivo* mineralocorticoid potency is ∼1/10–1/40; sustained elevations (e.g. DOC-producing tumors) can still drive apparent mineralocorticoid excess (27). Transient, modest 11-DOC rises under selective CYP11B2 inhibition are plausibly buffered by cortisol competition at the MR and by 11β-HSD2-mediated prereceptor metabolism, consistent with the absence of hyperkalemia observed with baxdrostat despite measurable increases in DOC (25, 28).

Precursor redistribution further distinguished between the agents. Baxdrostat largely spared upstream Δ5/Δ4 pathways (modest shifts in 17-OHP and DHEA-S). By contrast, osilodrostat increased androgens (e.g. androstenedione/testosterone), consistent with upstream accumulation and peripheral conversion, raising concerns about clinical sequelae (hirsutism/acne) in susceptible populations. Cross-tissue analyses strengthened generalizability: osilodrostat cortisol IC_50_ was right-shifted in CPT cultures with autonomous cortisol production vs APA, while baxdrostat remained non-inhibitory for cortisol over the tested range. A practical pharmacodynamic marker emerged, the 11-DOC/cortisol ratio, which increased modestly with baxdrostat (mean FC ∼ 1.8) and substantially with osilodrostat (∼12.5), quantitatively reflecting selectivity and offering a candidate readout for dose optimization and on-target monitoring in future trials.

### Therapeutic positioning

MRAs remain effective for BP and organ protection but are constrained by hyperkalemia, antiandrogenic effects, and aldosterone escape (3, 4, 8). Even with newer nonsteroidal receptor-level agents (e.g. finerenone) (29), source-level aldosterone suppression offers a mechanistic advantage and may afford cardio-renal benefits beyond BP lowering, given aldosterone-driven inflammation, fibrosis, and endothelial dysfunction (1, 2). Our *ex vivo* clinical concordance supports baxdrostat as a highly selective ASI that reduces aldosterone while preserving the cortisol axis, consistent with observations in resistant hypertension and primary aldosteronism (16, 17, 26). Selective ASIs therefore represent a meaningful advance in targeted antihypertensive therapy. Beyond resistant hypertension, selective ASIs may benefit HFpEF, chronic kidney disease, and primary aldosteronism (definitive therapy or pre-surgical bridging) (30, 31). Future work should test rational combinations (e.g. ASI + SGLT2 inhibition), evaluate predictive biomarkers (e.g. baseline PRA, CYP11B variants), and pursue long-term outcome trials to position ASIs within precision hypertension algorithms.

To further investigate the mechanism of action, additional RT-qPCR analyses were conducted on four newly established primary culture samples following 72 h of exposure to baxdrostat or osilodrostat. The stability of RPL32 expression across treatment groups was verified prior to analysis (mean ± SD = 18.90 ± 0.40 cycles, *n* = 36; one-way ANOVA, *P* > 0.95 for both PCR runs). No consistent pattern of transcriptional downregulation for CYP11B1, CYP11B2, or CYP17A1 was observed across donors (Supplementary Fig. 15). For CYP17A1, the magnitude of FCs in informative cases was within the pre-specified ±2-fold equivalence bound (TOST P_equiv ≤ 0.04). By contrast, for CYP11B1 and CYP11B2, the point estimates of FC remained within ±2-fold relative to vehicle controls; however, the 90% CIs were broader owing to limited sample size and greater technical variability associated with low-abundance transcripts. As a result, formal transcriptional invariance cannot be asserted for these targets. Notably, changes in steroid levels measured by LC–MS/MS, specifically the three- to five-fold suppression of aldosterone or cortisol secretion, were substantially greater than the corresponding changes at the mRNA level (Supplementary Fig. 16). These findings support a predominantly enzyme-inhibitory rather than transcriptional mechanism during this 72-h exposure window.

### Limitations

First, the sample size was modest, particularly NAG (*n* = 3), and not all assays were completed across donors. Second, analyses were designed for pharmacologic characterization rather than prediction; therefore, we report effect sizes with 95% CIs and mixed-effects estimates. Third, the 72-h treatment duration employed in this study represents only short-term exposure and does not account for longer-term adaptive responses in steroidogenesis, including enzyme induction, cellular hypertrophy or atrophy, depletion of intracellular steroid reserves, or escape mechanisms that may arise during chronic treatment. Fourth, *ex vivo* → *in vivo* translation warrants caution because protein binding, metabolism, tissue distribution, and endocrine feedback (ACTH/RAAS) are not modeled. Fifth, our *in vitro* system does not fully replicate the complex endocrine regulatory environment *in vivo*, including dynamic ACTH fluctuations and their interactions with the renin–angiotensin–aldosterone system. Since our experiments were conducted under a single stimulation condition, employing continuous ACTH (10 nM), the selectivity of ASIs in response to other hormonal stimuli, such as angiotensin II or potassium, remains to be elucidated. Sixth, while our molecular docking simulations suggest a plausible structural rationale for baxdrostat selectivity, these computational models require experimental verification. Notably, the predicted selectivity profiles align with previously reported biochemical and functional findings demonstrating selective CYP11B2 inhibition by baxdrostat (13) and dual CYP11B1/CYP11B2 inhibition by osilodrostat (32). Seventh, the additional RT-qPCR analyses were performed on a limited set of donors; consequently, these results must be interpreted descriptively rather than as conclusive evidence of transcriptional invariance. Furthermore, potential long-term adaptive transcriptional modifications arising from chronic exposure cannot be excluded based on the current data. Eighth, tumor heterogeneity may increase variability and obscure small effects. Finally, the proposed 11-DOC/cortisol ratio is exploratory and requires prospective validation versus BP outcomes before clinical implementation.

## Conclusion

Head-to-head studies in primary human adrenocortical cells (APA/CPT/NAG) show that baxdrostat selectively inhibits CYP11B2 while largely sparing CYP11B1-dependent cortisol synthesis, whereas osilodrostat exerts less selective dual CYP11B2/CYP11B1 inhibition. Convergent functional, LC–MS/MS, and docking data link CYP11B2 active-site engagement to the selective steroidogenic signature of baxdrostat. These findings support further clinical evaluation of baxdrostat and other highly selective ASIs for aldosterone-mediated hypertension and motivate longer-term studies with prospective biomarker validation (e.g. 11-DOC/cortisol) and combination strategies (e.g. ASI + RAAS or SGLT2 inhibition) to optimize precision therapy.

## Supplementary materials











## Declaration of interest

The authors declare that there is no conflict of interest that could be perceived as prejudicing the impartiality of the research reported.

## Funding

This work was supported by grants from the Ministry of Education, Culture, Sports, Science and Technology (Japan) (Grants-in-Aid: for Scientific Research (B) #22300325, #19H03708, #21H02974, #23H02809, #23K24147; (C) #25K11780, #25K10723, #24K10279, 23K09074, #22K07205, #22K08619; Challenging Research (Pioneering) and (Exploratory) #23K17429, #21K19398; JSPS Core-to-Core Program (JPJSCCA20200006)). This work was supported by the Japan Science and Technology Agency (JST) (grant no. JPMJPF2301). This work was partly supported by the Uehara Memorial Foundation, the Mochida Memorial Foundation for Medical and Pharmaceutical Research, the Naito Foundation, the Mitsui Life Social Welfare Foundation, the Princes Takamatsu Cancer Research Fund, the Takeda Science Foundation, and the Senshin Medical Research Foundation.

## Author contribution statement

YT contributed to conceptualization, methodology, investigation, formal analysis, and writing of the original draft. TK contributed to conceptualization, formal analysis, writing of the original draft, and review and editing. IS, NH, and MY contributed to supervision and review and editing. YN and HS contributed to investigation (immunohistochemistry) and data curation. EK and TM contributed to formal analysis (statistics) and methodology. TT contributed to conceptualization, methodology, funding acquisition, project administration, supervision, and review and editing. All authors approved the final manuscript, and TT is the guarantor.

## Data availability

The data underlying this study are available from the corresponding author on reasonable request.

## Study design and setting

This was an *in vitro* experimental study using primary human adrenocortical cells. All experiments were conducted at the Department of Molecular Diagnosis, Graduate School of Medicine, Chiba University.

## Ethics

This study was approved by the Institutional Review Board (IRB: HS202204-06) of Chiba University Hospital. Written informed consent was obtained from all participants, and procedures complied with the Declaration of Helsinki.
