# Supplementary material for: Baxdrostat versus osilodrostat: steroid biosynthesis in human adrenocortical cells
Source: Endocr Connect. 2026 Jul 15;15(7):e250807. doi: 10.1530/EC-25-0807 (PMC13383238; doi:10.1530/EC-25-0807)
Supplement: Supplementary file 1 [file EC-25-0807_supplementary_figures_1-3.pdf]

Supplementary Fig 1

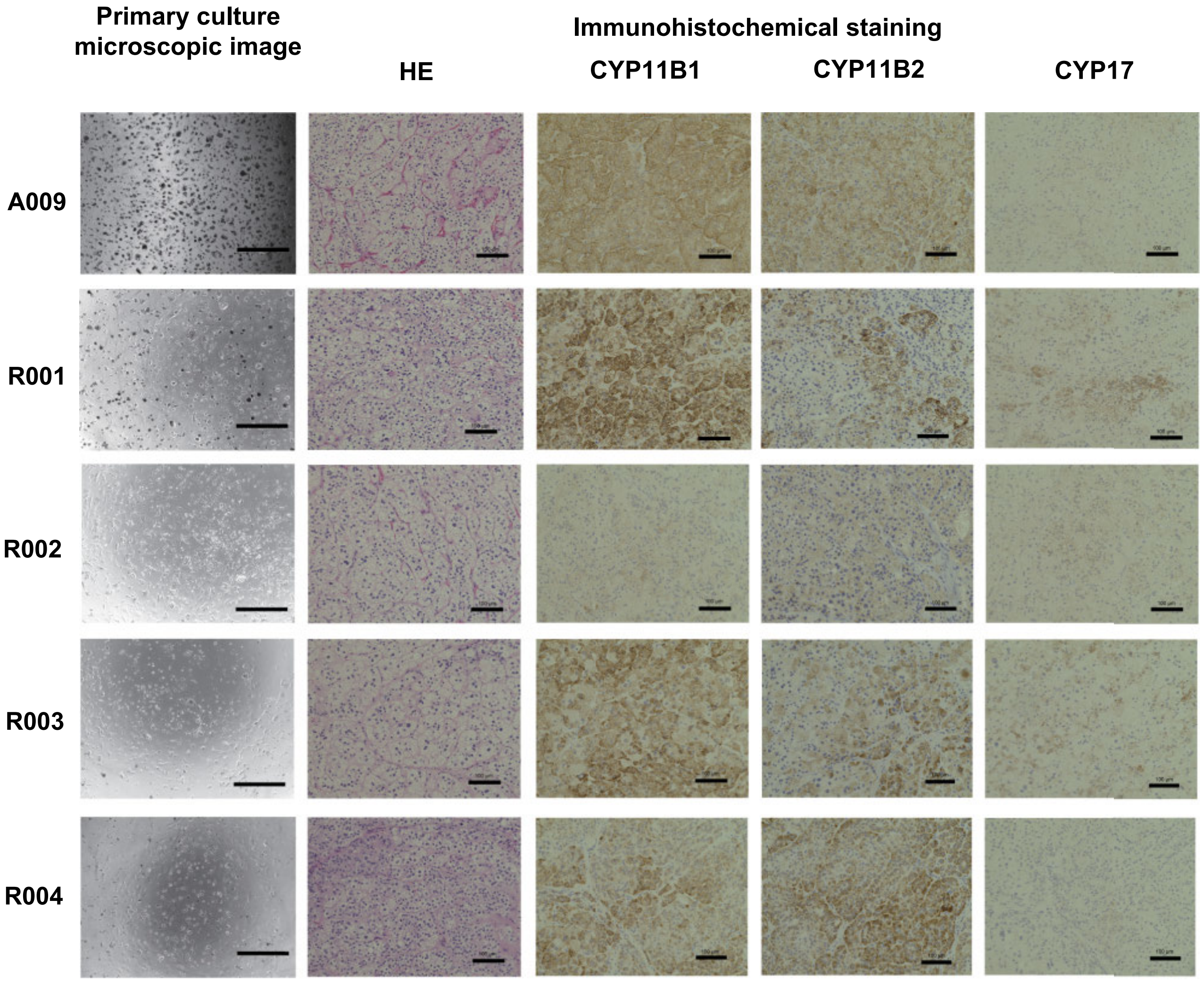

Supplementary Figure 1. Representative Histopathological and immunohistochemical Images of Additional APA Specimens

Representative brightfield images of primary cultures and corresponding histological sections from aldosterone-producing adenomas (A009, R001, R002, R003, R004). Hematoxylin and eosin (HE) staining and immunohistochemical staining for CYP11B2 (aldosterone synthase), CYP11B1 (11β-hydroxylase), and CYP17A1 (17α-hydroxylase/17,20-lyase) are shown. Scale bars: 750 µm (brightfield), 100 µm (immunohistochemistry).

Abbreviations: APA, aldosterone-producing adenoma; HE, hematoxylin and eosin.

Supplementary Fig 2

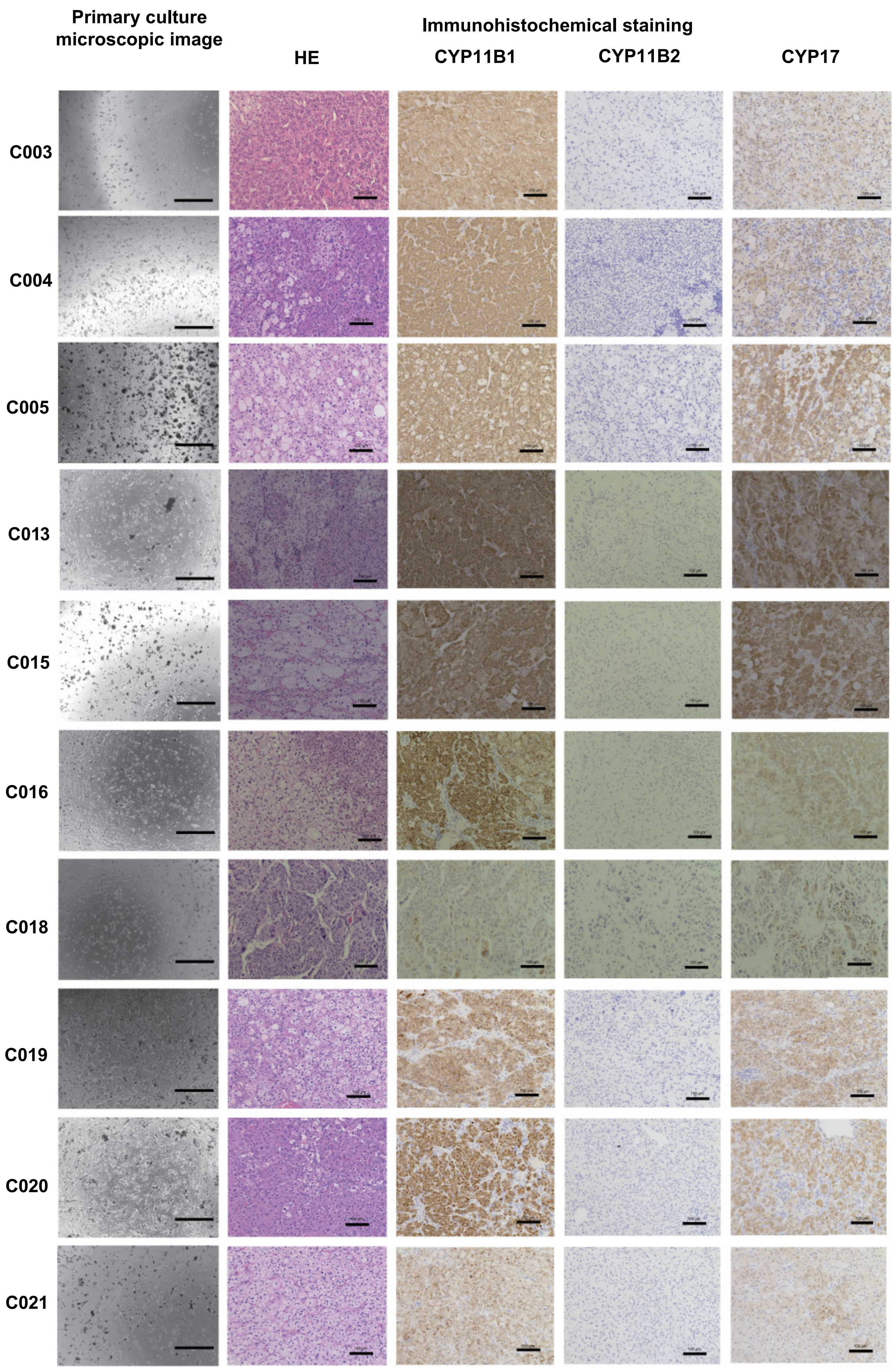

Supplementary Figure 2. Representative Histopathological and Immunohistochemical Images of CPT Specimens

Representative brightfield images of primary cultures and corresponding histological sections from cortisol-producing tumors (C003, C004, C005, C013, C015, C016, C018, C019, C020, C021). HE staining and immunohistochemical staining for CYP11B2, CYP11B1, and CYP17A1 demonstrate the expression patterns of steroidogenic enzymes in cortisol-producing tissue. Scale bars: 750 µm (brightfield), 100 µm (immunohistochemistry).

Abbreviations: CPT, cortisol-producing tumor; HE, hematoxylin and eosin.

Supplementary Fig 3

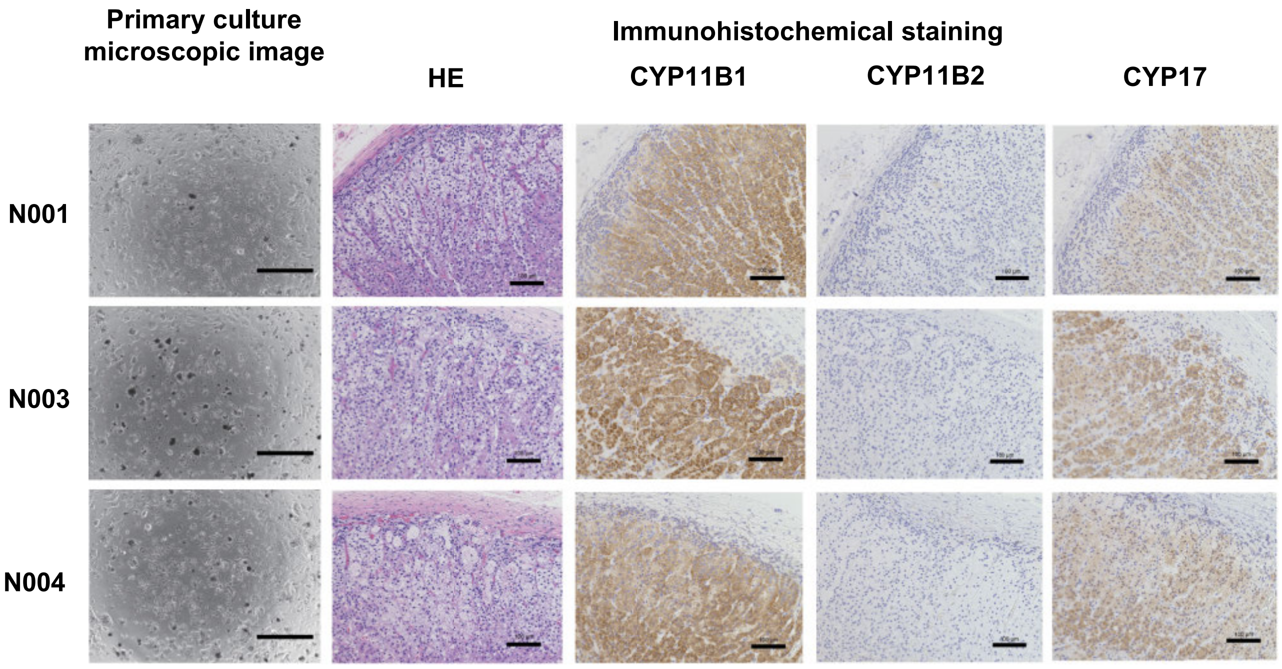

Supplementary Figure 3. Representative Histopathological and Immunohistochemical Images of NAG Specimens

Representative brightfield images of primary cultures and corresponding histological sections from normal adrenal glands (N001, N003, N004). HE staining and immunohistochemical staining for CYP11B2, CYP11B1, and CYP17A1 show normal expression patterns in non-tumorous adrenal cortex. Scale bars: 750 µm (brightfield), 100 µm (immunohistochemistry).

Abbreviations: NAG, normal adrenal gland; HE, hematoxylin and eosin.
