# Supplementary material for: Baxdrostat versus osilodrostat: steroid biosynthesis in human adrenocortical cells
Source: Endocr Connect. 2026 Jul 15;15(7):e250807. doi: 10.1530/EC-25-0807 (PMC13383238; doi:10.1530/EC-25-0807)
Supplement: Supplementary file 2 [file EC-25-0807_supplementary_figures_4_5.pdf]

Supplementary Fig 4

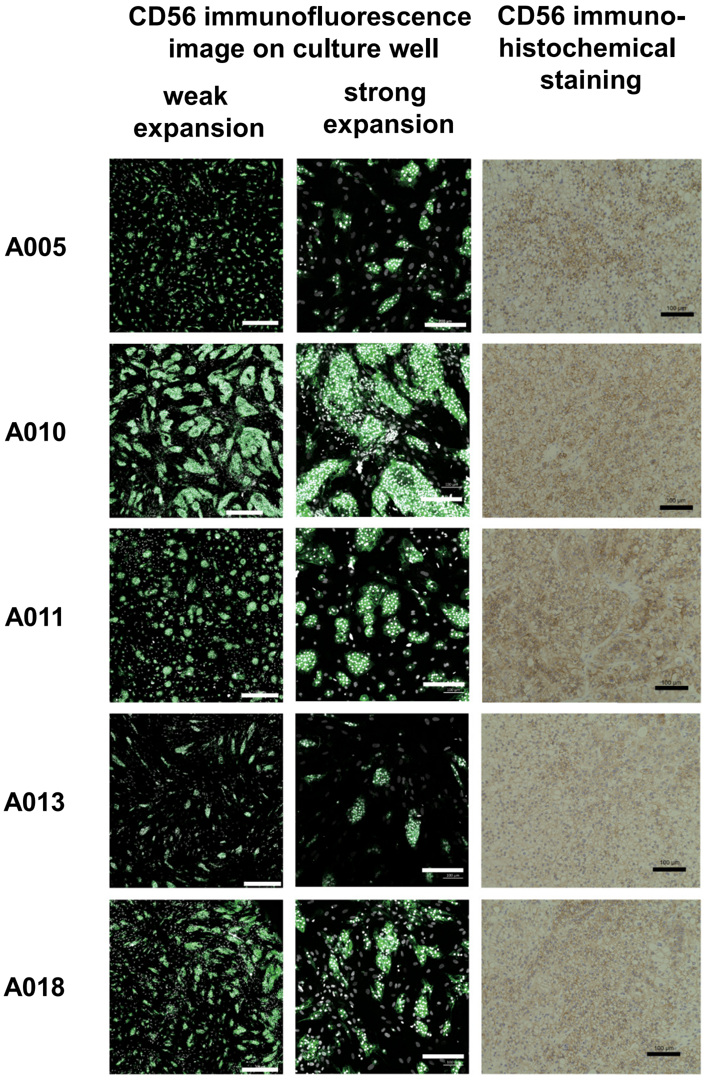

Supplementary Figure 4. CD56 Immunofluorescence Characterization of APA-Derived Primary Cultures

CD56 immunofluorescence images of primary adrenocortical cultures derived from aldosterone-producing adenomas (A005, A010, A011, A013, A018), showing cells with weak and strong expansion patterns. Corresponding CD56 immunohistochemical staining of the original tissue sections is shown for comparison. CD56 serves as a marker for human adrenocortical cells and is used to confirm the adrenocortical origin of cultured cells. Scale bars: 500 µm (weak expansion CD56 immunofluorescence image), 200 µm (strong expansion CD56 immunofluorescence image), 100 µm (CD56 immunohistochemical image).

Abbreviations: APA, aldosterone-producing adenoma.

Supplementary Fig 5

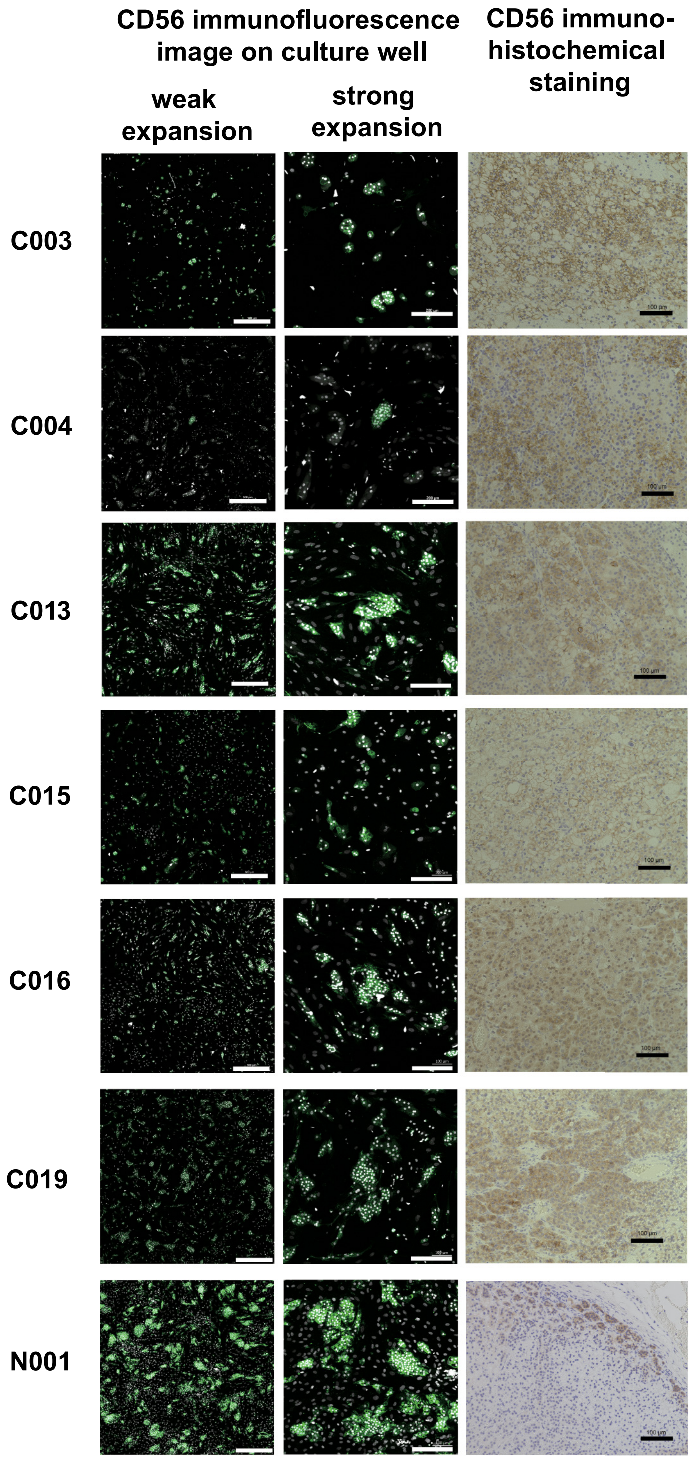

Supplementary Figure 5. CD56 Immunofluorescence Characterization of CPT and NAG-Derived Primary Cultures

CD56 immunofluorescence images of primary adrenocortical cultures derived from cortisol-producing tumors (C003, C004, C013, C015, C016, C019) and normal adrenal gland (N001), displaying cells with varying expansion patterns. Corresponding CD56 immunohistochemical staining demonstrates the heterogeneity of adrenocortical cell populations in different tissue types. Scale bars: 500 μm (weak expansion CD56 immunofluorescence image), 200 μm (strong expansion CD56 immunofluorescence image), 100 μm (CD56 immunohistochemical image).

Abbreviations: CPT, cortisol-producing tumor; NAG, normal adrenal gland.
