# Supplementary material for: Baxdrostat versus osilodrostat: steroid biosynthesis in human adrenocortical cells
Source: Endocr Connect. 2026 Jul 15;15(7):e250807. doi: 10.1530/EC-25-0807 (PMC13383238; doi:10.1530/EC-25-0807)
Supplement: Supplementary file 3 [file EC-25-0807_supplementary_figures_6-16.pdf]

Supplementary Fig 6

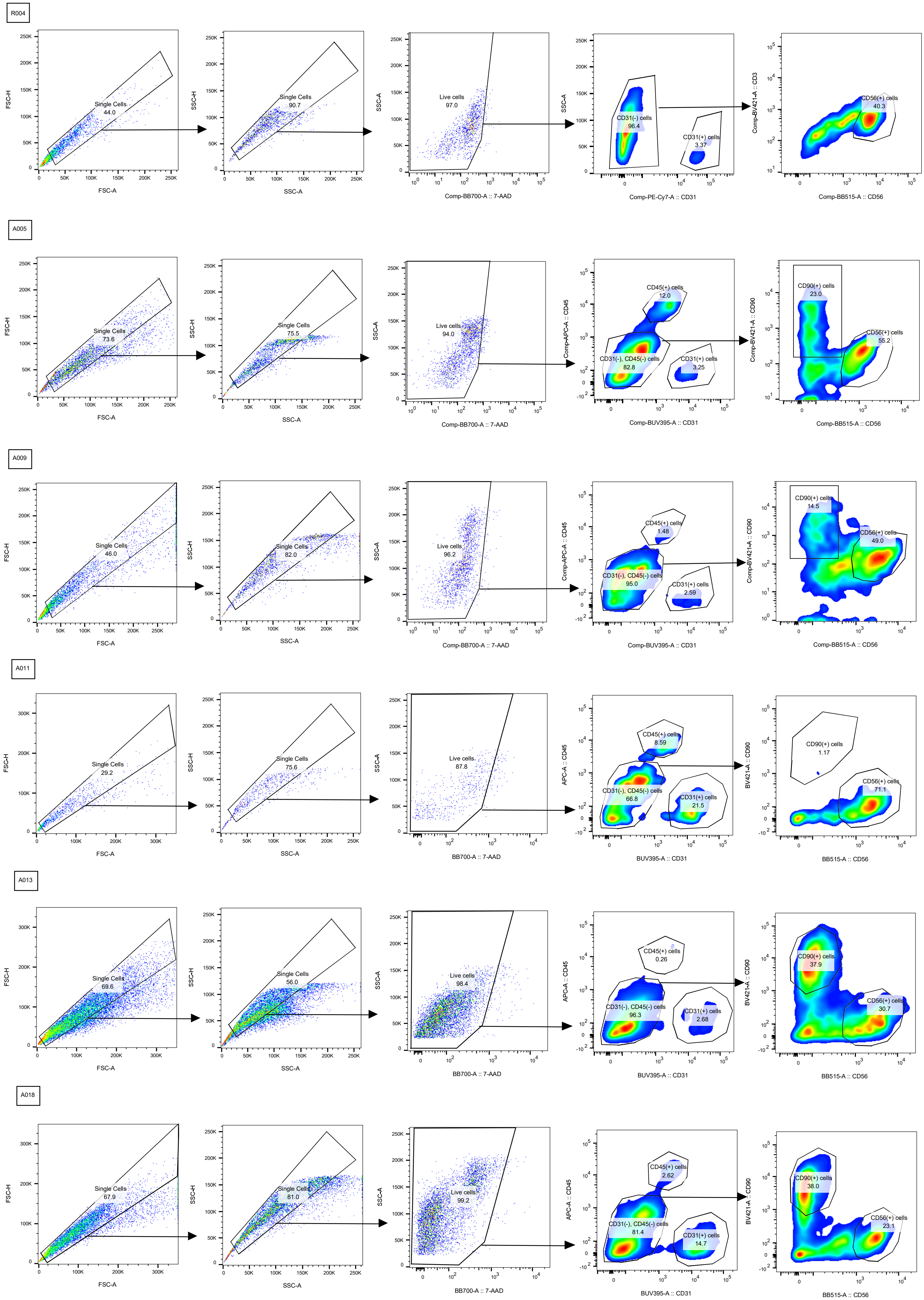

Supplementary Fig. 6. Flow cytometric gating strategy for primary adrenocortical cell cultures derived from aldosterone-producing adenomas (APA).

Each row shows the sequential FACS gating strategy applied to an independent APA donor. Cells were sequentially gated for single cells (FSC-A vs SSC-A and FSC-A vs FSC-H), viable cells (7-AAD–negative), separation of CD45-negative adrenocortical cells from CD45-positive haematopoietic cells, and identification of viable CD56-positive adrenocortical cells (CD45-/CD56+). Numbers within each gate indicate the percentage of cells in the parent population. Sample identifiers are shown at the upper-left corner of each row. These data confirm enrichment of CD56-positive adrenocortical cells in the primary cultures and underpin the quantitative summary presented in the main flow-cytometry results.

Supplementary Fig 7

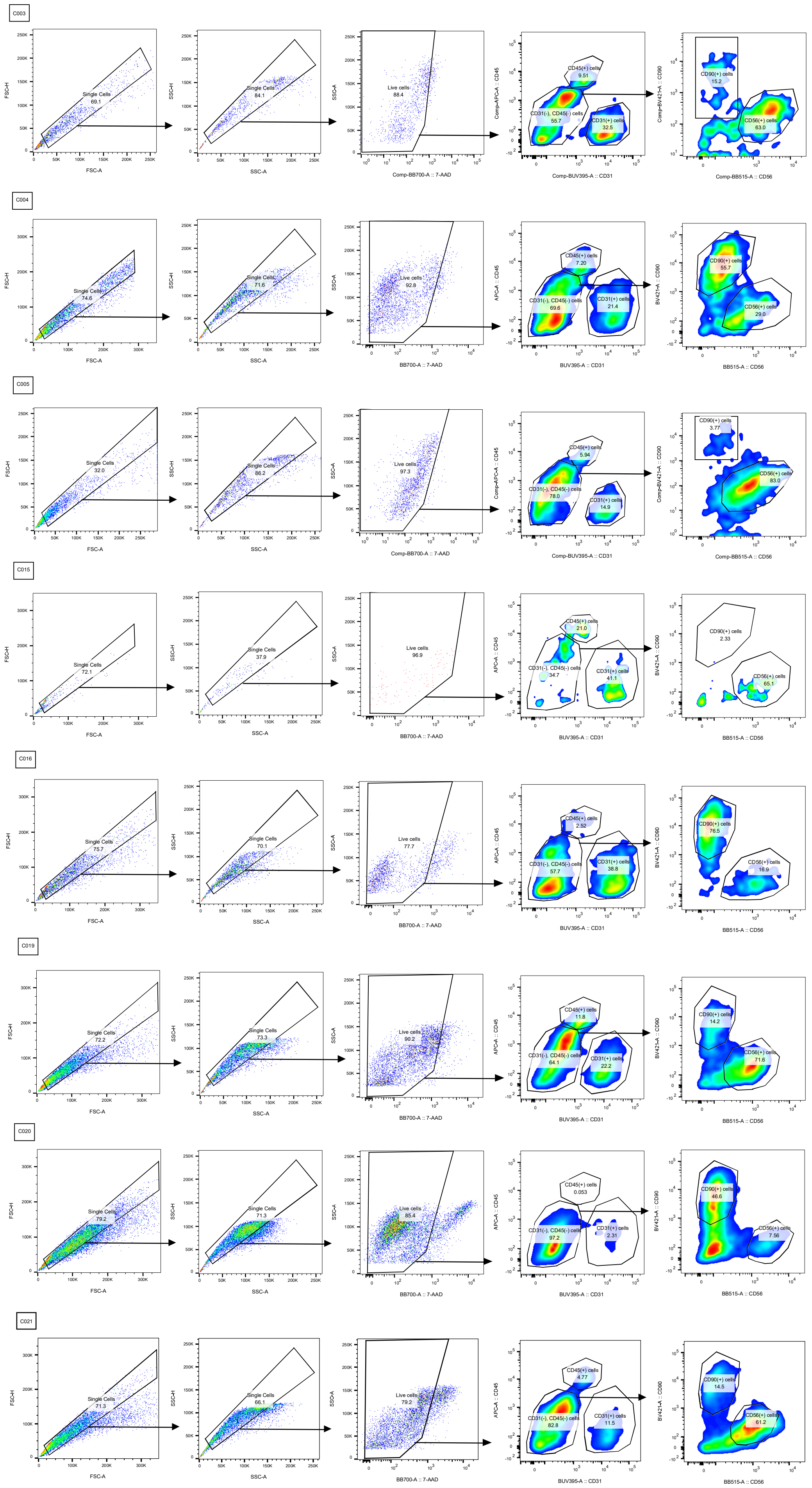

Supplementary Fig. 7. Flow cytometric gating strategy for primary adrenocortical cell cultures derived from cortisol-producing tumors (CPT).

Each row shows the sequential FACS gating strategy applied to an independent CPT donor, following the same workflow as Supplementary Fig. 6: single-cell selection, 7-AAD-based live-cell gating, separation of CD45-negative adrenocortical cells from CD45-positive haematopoietic cells, and identification of viable CD56-positive adrenocortical cells. Sample identifiers are shown at the upper-left corner of each row. Percentages within each gate indicate the proportion of cells in the parent population, supporting the quality and identity of the CPT primary cultures.

Supplementary Fig 8

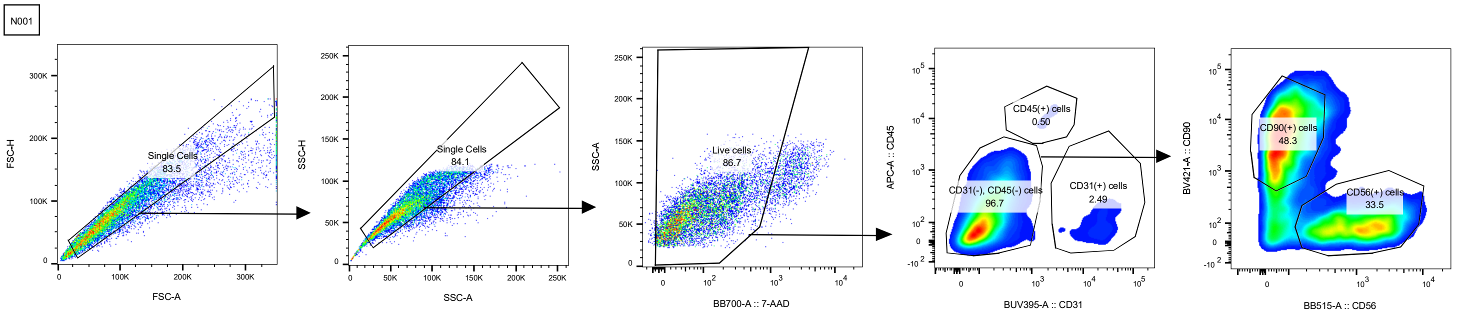

Supplementary Fig. 8. Flow cytometric gating strategy for primary adrenocortical cell cultures derived from histologically normal adrenal cortex (NAG).

Representative FACS gating strategy applied to a normal adrenal gland (NAG) donor obtained from adrenocortical tissue adjacent to a pheochromocytoma. The same sequential gating workflow as in Supplementary Figs. 6 and 7 is applied: single-cell selection (FSC-A vs SSC-A; FSC-A vs FSC-H), 7-AAD-based viability gating, CD45-/CD45+ separation, and CD56-positive adrenocortical cell identification. Percentages within each gate indicate the proportion of cells in the parent population.

Supplementary Fig 9

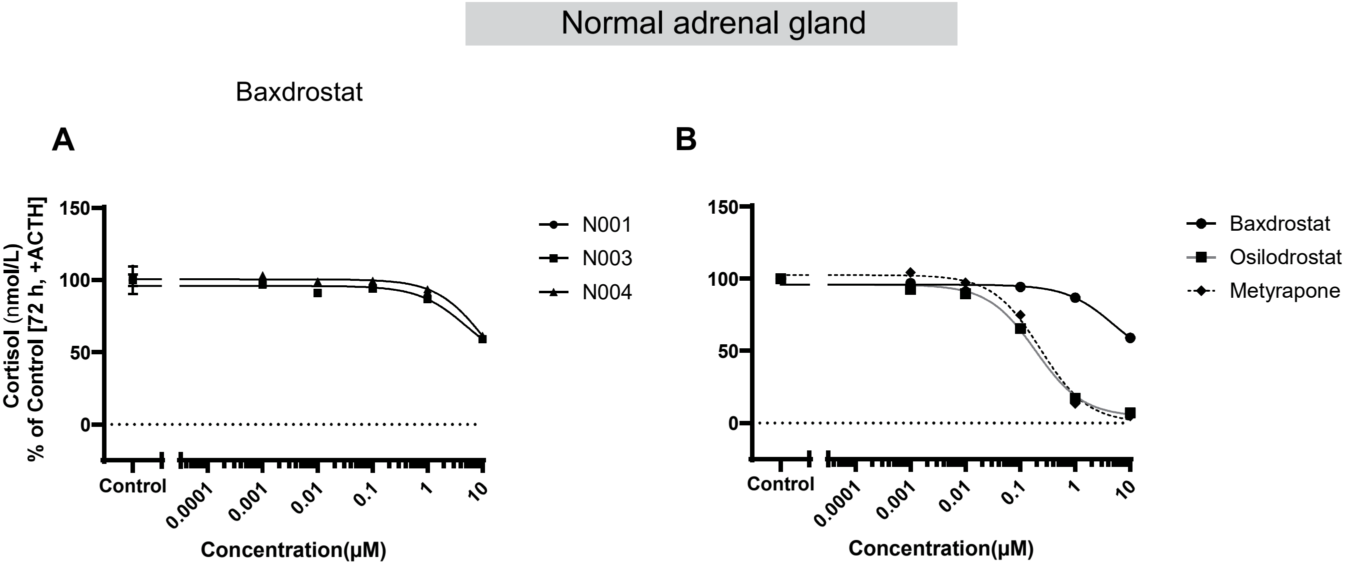

Supplementary Fig. 9. Cortisol dose-response of CYP11B inhibitors in normal adrenal gland (NAG) primary cultures.

(A) Per-donor cortisol concentration-response curves for baxdrostat across three NAG donors (N001, N003, N004) under continuous ACTH (10 nM) stimulation for 72 h. Cortisol levels are expressed as a percentage of vehicle control. (B) Concentration-response curves in N003 for baxdrostat, osilodrostat, and metyrapone. Baxdrostat did not reach 50% cortisol inhibition within the 0-10 μM range, consistent with its CYP11B2-selective profile, whereas osilodrostat and metyrapone produced substantial cortisol suppression. Curves were fit using a three-parameter logistic model (top fixed at 100%, bottom > or = 0).

Supplementary Fig 10

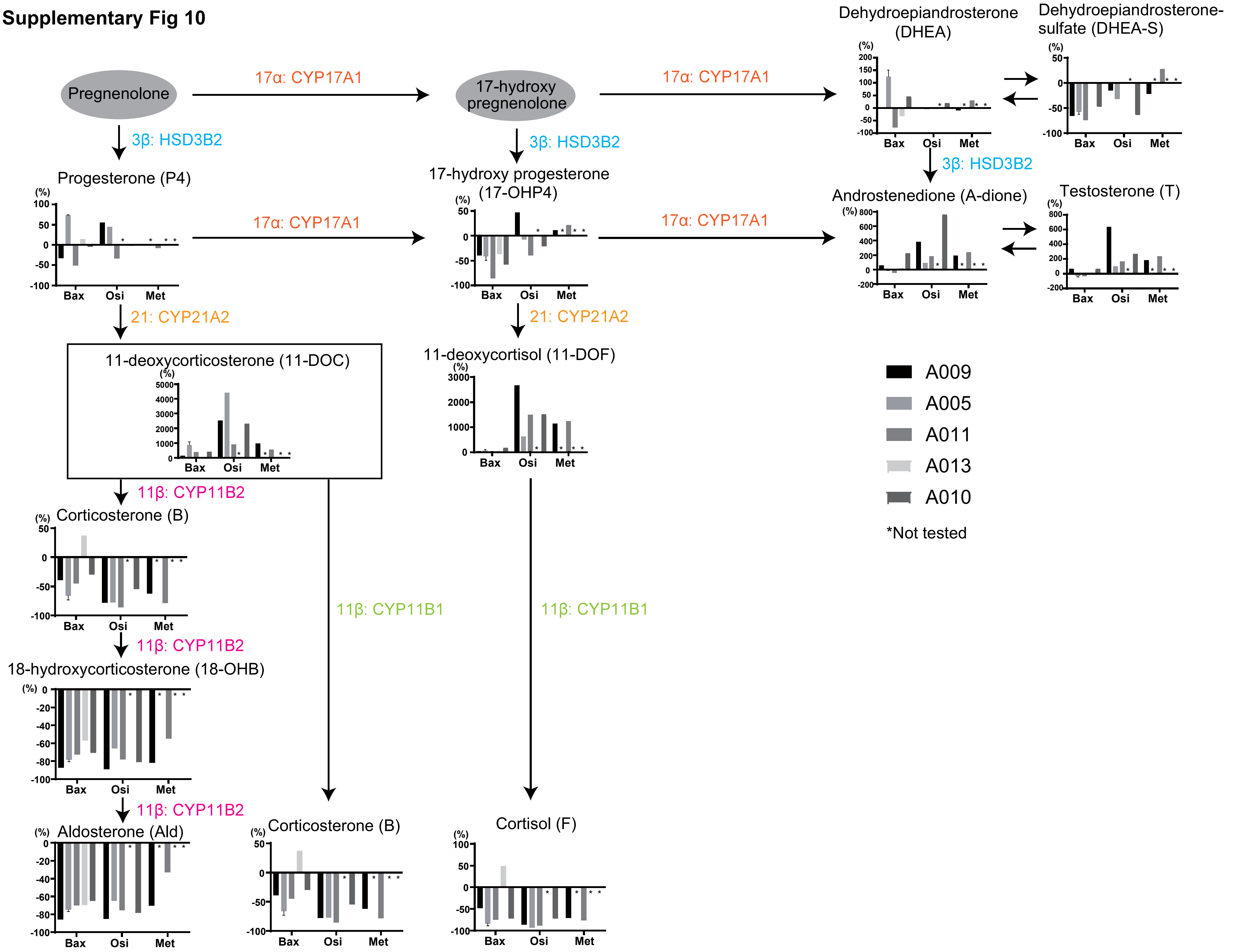

Supplementary Fig. 10. Steroidogenic-pathway profiling of CYP11B inhibitors in APA primary cultures.

Schematic of the steroidogenic pathway, overlaid with bar plots showing per-donor percent change versus vehicle for each detected steroid metabolite in aldosterone-producing adenoma (APA) primary cultures after 72-h treatment with baxdrostat (Bax), osilodrostat (Osi), or metyrapone (Met) at suprapharmacological concentrations (1 μM). Bars correspond to individual donors (A009, A005, A011, A013, A010), as indicated in the legend. Asterisks ("*Not tested") indicate samples not analyzed for a particular metabolite. Enzymes catalyzing each step are color-coded (17α: CYP17A1; 3β: HSD3B2; 21: CYP21A2; 11β: CYP11B2/ CYP11B1).

Supplemental Fig 11

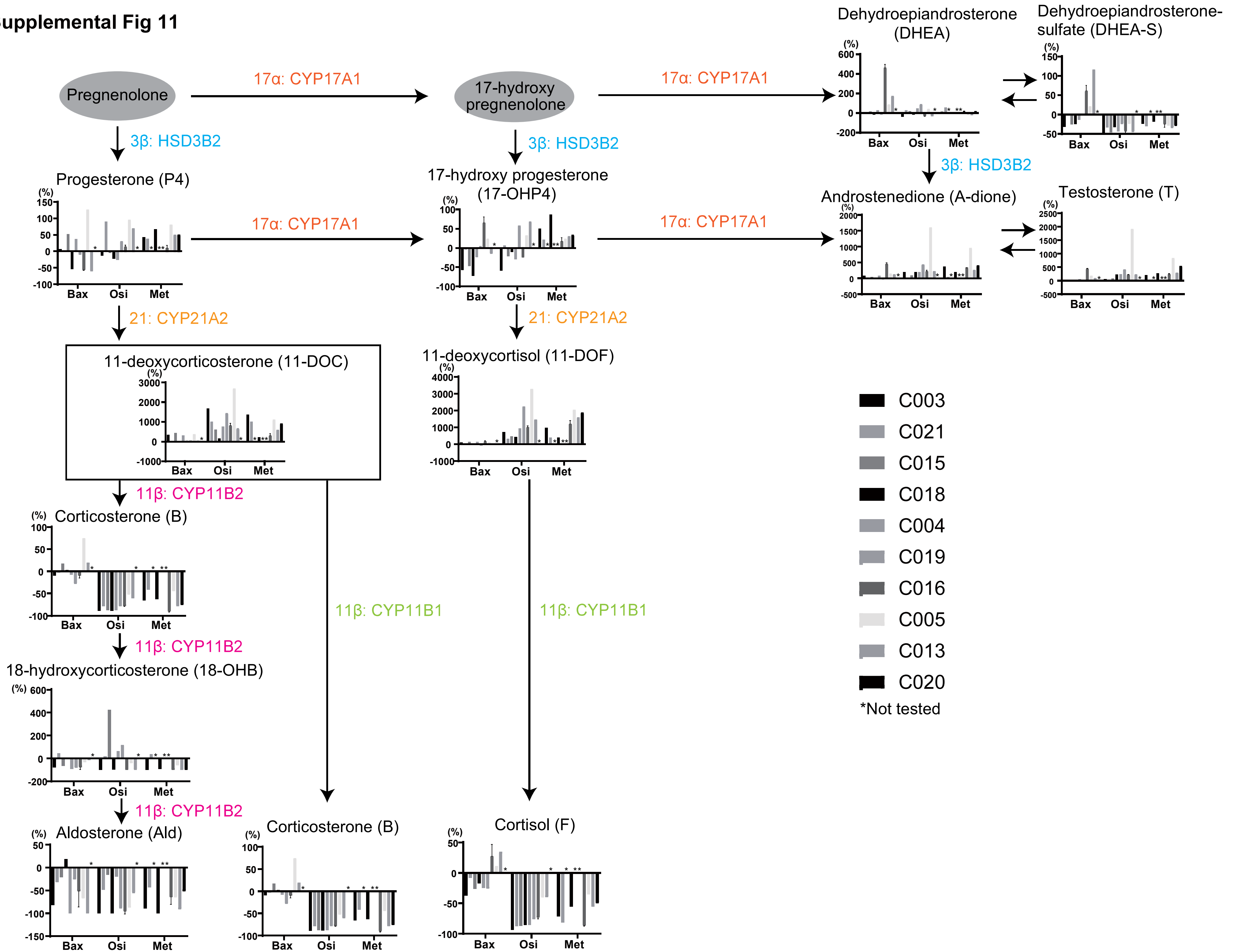

Supplementary Fig. 11. Steroidogenic-pathway profiling of CYP11B inhibitors in CPT primary cultures.

Schematic of the steroidogenic pathway, overlaid with bar plots showing per-donor percent change versus vehicle for each detected steroid metabolite in cortisol-producing tumor (CPT) primary cultures after 72-h treatment with baxdrostat (Bax), osilodrostat (Osi), or metyrapone (Met) at 1 μM. Bars correspond to individual donors (C003, C021, C015, C018, C004, C019, C016, C005, C013, C020), as indicated in the legend. Asterisks ("*Not tested") indicate samples not analyzed for a particular metabolite. Enzymes catalyzing each step are color-coded as in Supplementary Fig. 10.

Supplementary Fig 12

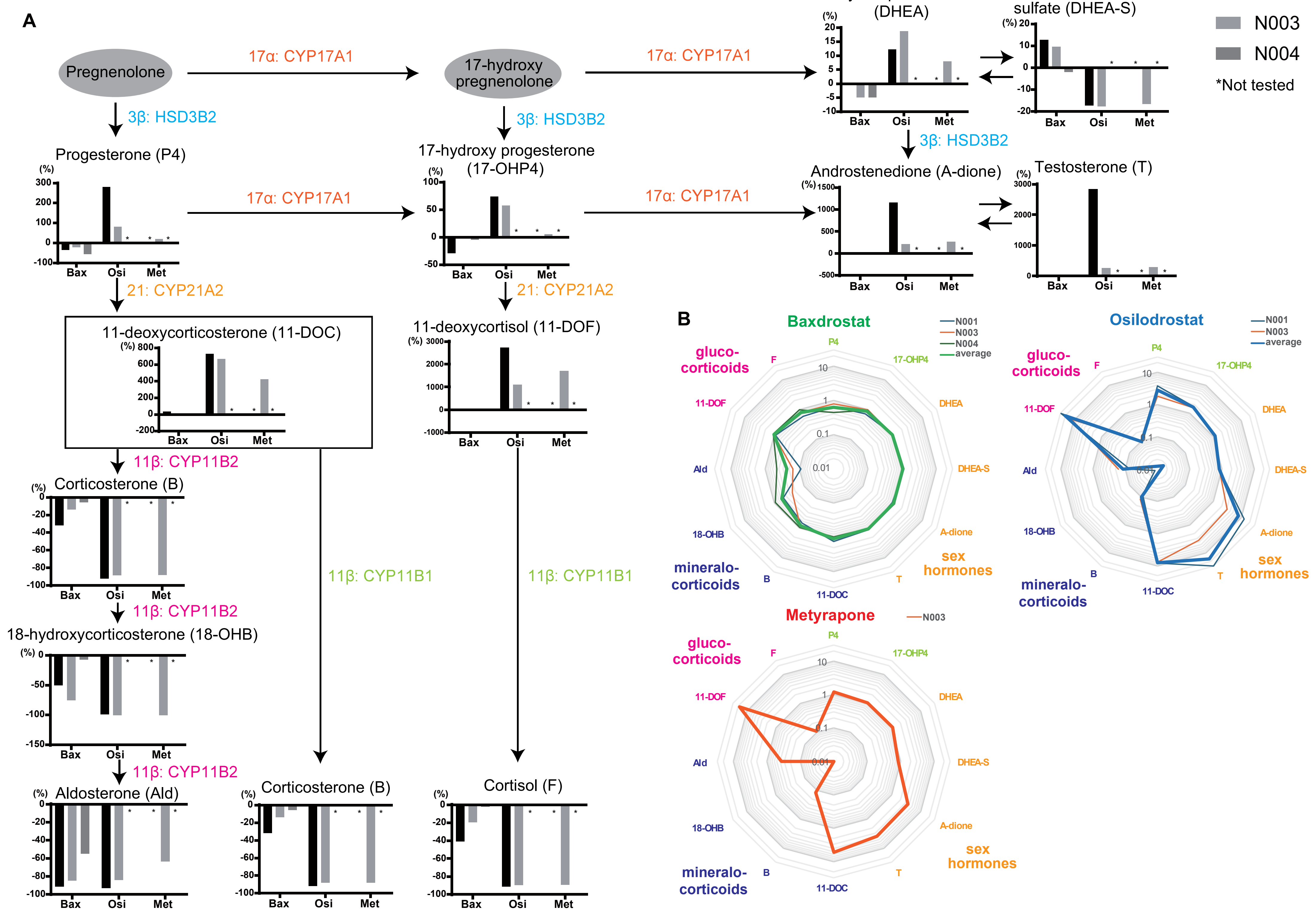

Supplementary Fig. 12. Steroidogenic-pathway profiling and radar summary of CYP11B inhibitors in NAG primary cultures.

(A) Schematic of the steroidogenic pathway, overlaid with bar plots showing per-donor percent change versus vehicle for each detected steroid metabolite in normal adrenal gland (NAG) primary cultures after 72-h treatment with baxdrostat (Bax), osilodrostat (Osi), or metyrapone (Met) at 1 µM. Bars correspond to individual donors (N001, N003, N004). (B) Radar plots summarizing the steroidogenic footprint of each drug across the 12 measured metabolites, with mineralocorticoid, glucocorticoid, and sex-hormone branches color-coded. Individual donor traces and per-drug averages are shown. Asterisks ("*Not tested") indicate samples not analyzed for a particular metabolite.

Supplementary Fig 13

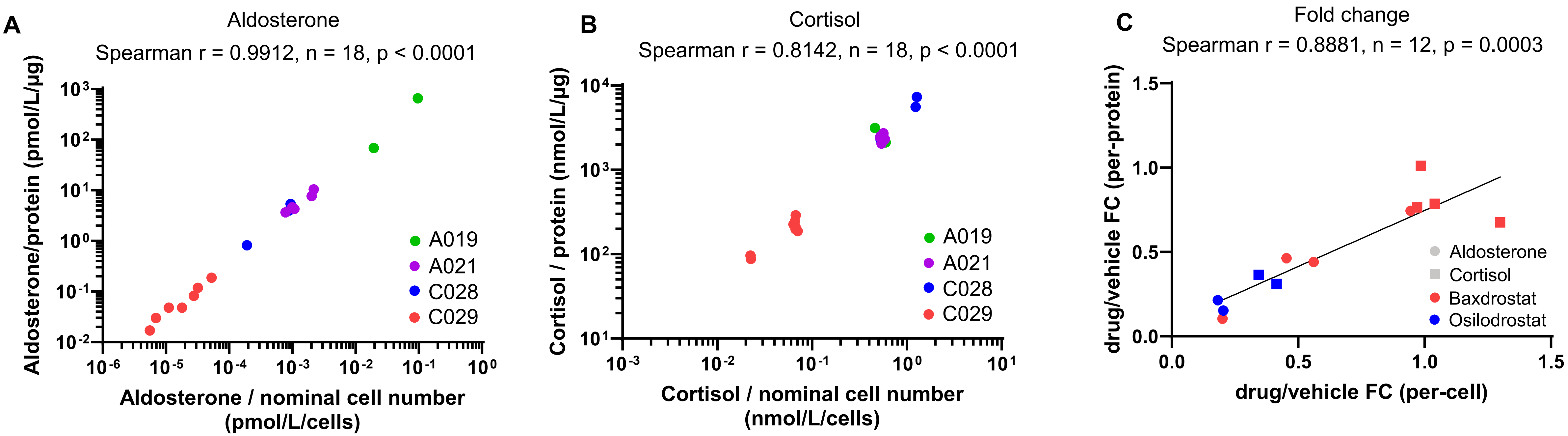

Supplementary Fig. 13. Validation of protein-normalized steroid output against normalization by assumed seeding cell number.

Validation in additional primary culture experiments using newly obtained adrenal tumor samples (two APA and two CPT cases). (A) Correlation between aldosterone normalized by the assumed seeding cell number (2×10^5 cells/well) and aldosterone normalized by total cellular protein quantified by BCA assay. (B) Same comparison for cortisol. (C) Concordance of drug/vehicle fold changes between the two normalization approaches; each point represents one case-drug-hormone combination. Spearman r values are computed for all panels. Each point in (A) and (B) represents a single well.

Supplementary Fig 14

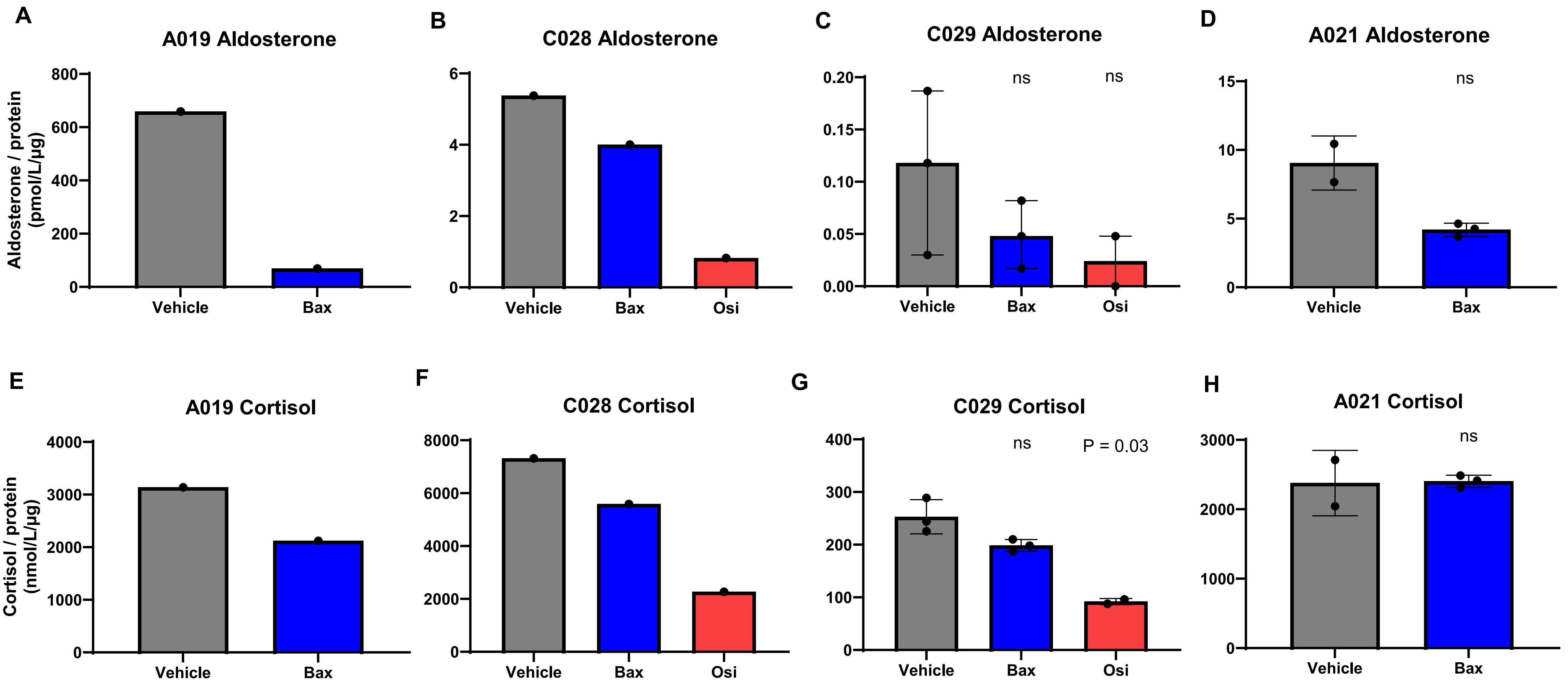

Supplementary Fig. 14. Protein-normalized hormone measurements in the additional primary culture experiments.

Primary cultures derived from A019 (APA), C028 (CPT), C029 (CPT), and A021 (APA) were treated with vehicle, baxdrostat (1 μM), or osilodrostat (1 μM), as available for each case. Panels (A-D) show aldosterone normalized to total cellular protein, and panels (E-H) show cortisol normalized to total cellular protein. Bars indicate median with interquartile range; dots indicate individual wells. Group differences were assessed by Kruskal-Wallis tests with Dunn post-hoc tests versus vehicle. P values represent exploratory well-level comparisons and were not intended as donor-level inference.

Supplementary Fig 15

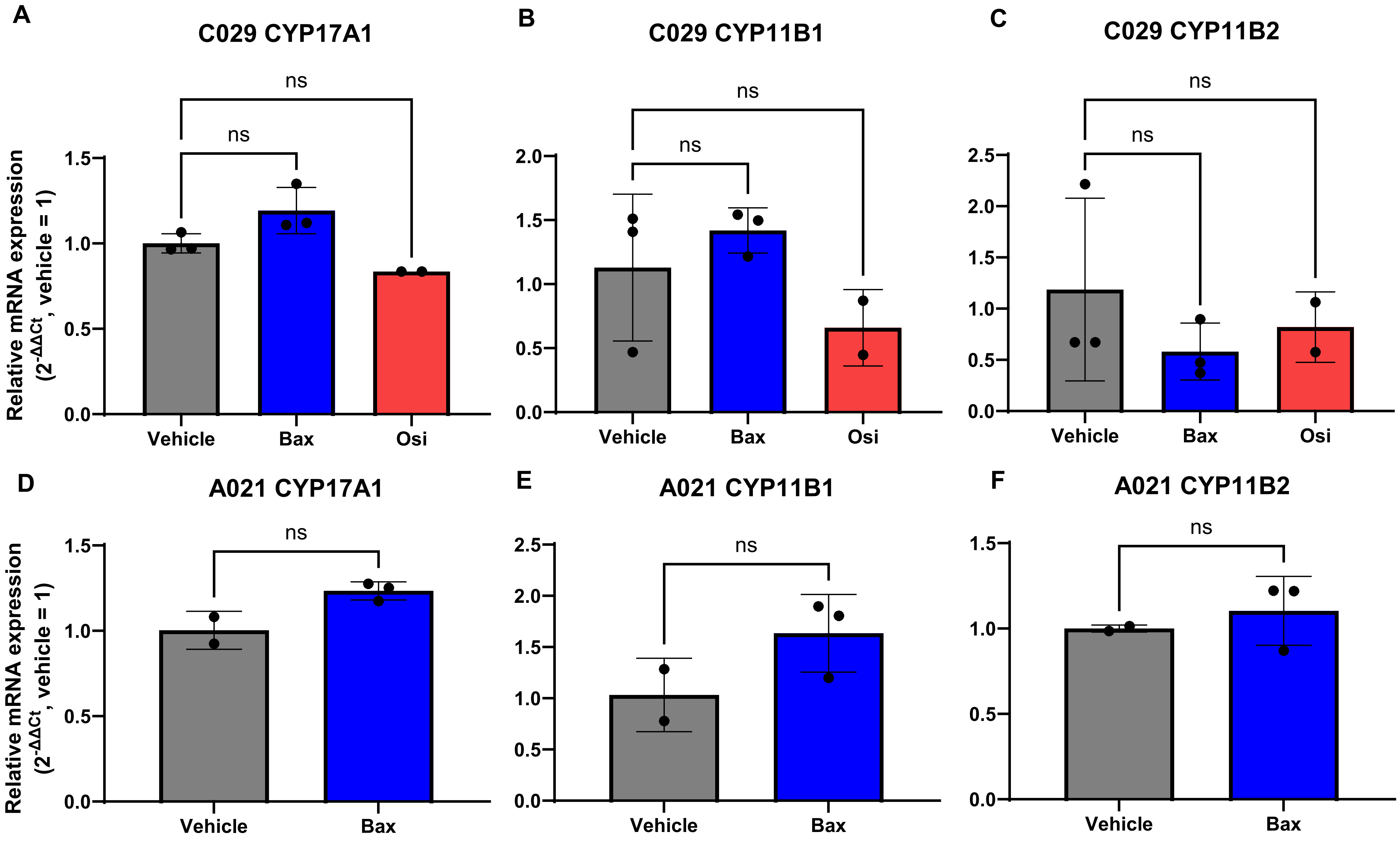

Supplementary Fig. 15. RT-qPCR analysis of steroidogenic-enzyme mRNA in informative cases.

Primary culture samples from C029 (CPT) and A021 (APA) were treated with vehicle, baxdrostat (1 μM), or osilodrostat (1 μM), as available for each case. Relative expression of CYP17A1, CYP11B1, and CYP11B2 was calculated as $2^{-\Delta\Delta Ct}$ using RPL32 as the internal reference gene and setting the mean value of the vehicle group within each case to 1. Bars indicate median with interquartile range; dots indicate individual wells. Informative cases were defined as those with ≥2 replicate wells per treatment group (C029: vehicle n = 3, baxdrostat n = 3, osilodrostat n = 2; A021: vehicle n = 2, baxdrostat n = 3). Statistical comparisons versus vehicle were performed using Kruskal-Wallis tests with Dunn post-hoc tests on ΔCt values (ns = not significant).

Supplementary Fig 16

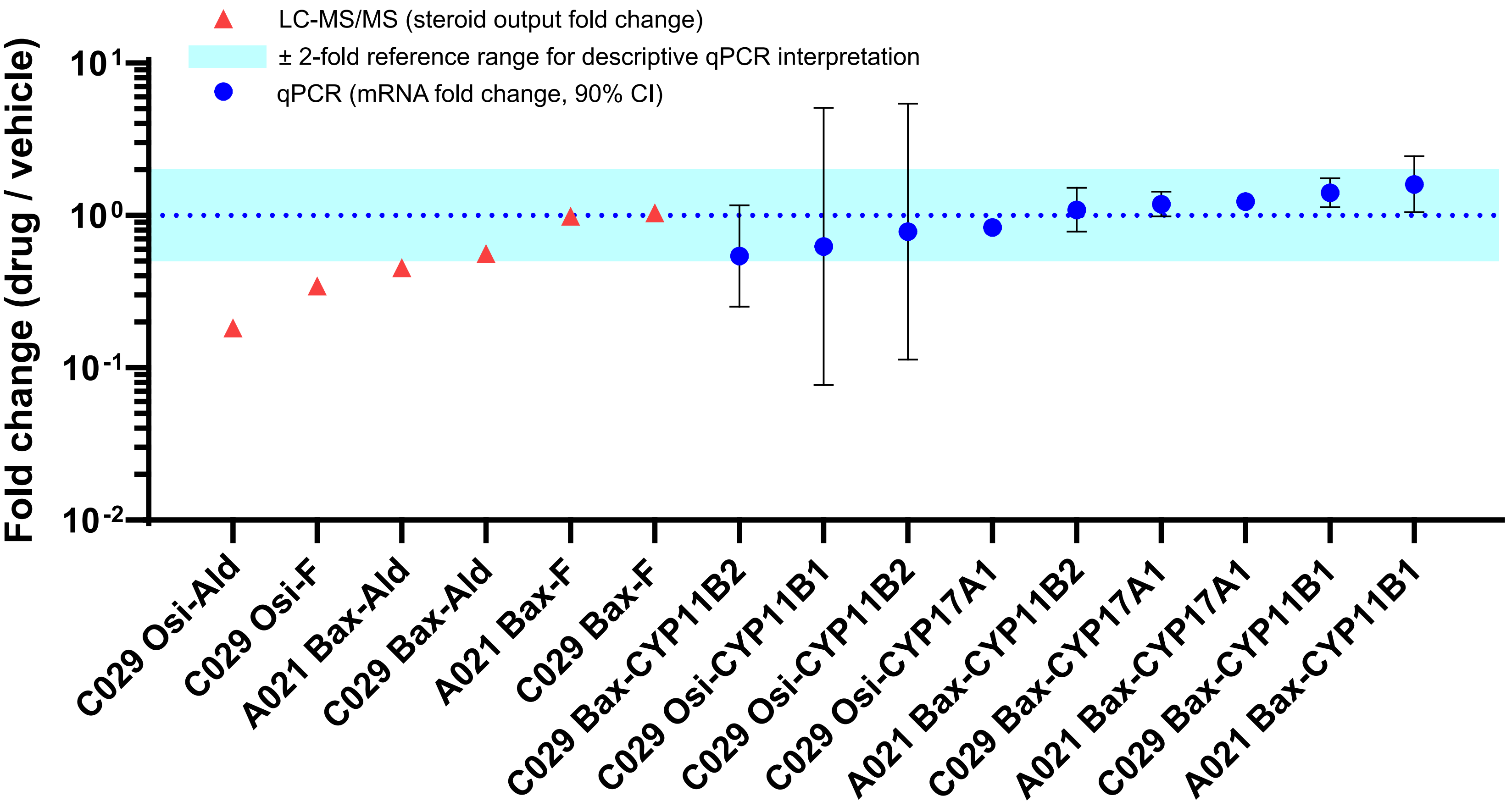

Supplementary Fig. 16. qPCR mRNA fold change vs LC–MS/MS steroid fold change.

Descriptive comparison of LC–MS/MS-derived steroid fold changes and RT-qPCR-derived mRNA fold changes in informative cases (C029 and A021). Red triangles indicate LC–MS/MS steroid-output fold changes (drug/vehicle) relative to the vehicle mean within each case. Blue circles indicate RT-qPCR fold changes for steroidogenic-enzyme mRNAs, shown as $2^{-\Delta\Delta Ct}$ relative to the case-specific vehicle mean, with error bars representing 90% confidence intervals for $\Delta\Delta Ct$-derived fold changes. The shaded region denotes the ±2-fold reference range used for descriptive qPCR interpretation. Overall, steroid output changed more markedly than steroidogenic-enzyme mRNA expression, consistent with a predominantly enzyme-inhibitory rather than transcriptional mechanism during the 72-hour exposure window.
