# Supplementary material for: Baxdrostat versus osilodrostat: steroid biosynthesis in human adrenocortical cells
Source: Endocr Connect. 2026 Jul 15;15(7):e250807. doi: 10.1530/EC-25-0807 (PMC13383238; doi:10.1530/EC-25-0807)
Supplement: Supplementary file 4 [file EC-25-0807_supplementary_materials.pdf]

Supplementary Methods

Ethics statement is provided in the main article.

1. Tissue dissociation and primary cell culture

Resected adrenal tissues were minced (~1–2 mm^3) and enzymatically dissociated at 37 °C for 90 min with collagenase type I (Worthington Biochemical Corp. (Lakewood, NJ, USA) LS004194) and collagenase type VII (Worthington Biochemical Corp. (Lakewood, NJ, USA) LS005332), followed by filtration (70 µm). Red cell lysis, when necessary, employed ACK lysing buffer (Thermo Fisher Scientific (Waltham, MA, USA), A1049201). Cells were seeded on Matrigel (Corning Inc. (Corning, NY, USA), 356237; growth factor–reduced) at 0.5–1 × 10^5 cells/well (24-well plate), in DMEM/F12 (Thermo Fisher Scientific (Waltham, MA, USA), 11320033) supplemented with 5% FBS (Thermo Fisher Scientific (Waltham, MA, USA), A5670201) and 1% penicillin–streptomycin (Thermo Fisher Scientific (Waltham, MA, USA), 15140122). After 72-96 h to allow attachment, the medium was replaced with fresh medium and experimental treatments were initiated.

2. Stimulation and inhibitor treatments

Unless indicated, cultures were stimulated with ACTH 10 nM (selected from pilot aldosterone/cortisol dose–response testing) and exposed for 72 h to baxdrostat, osilodrostat, or metyrapone across 0–10 µM (vehicle <0.1% DMSO). For LC–MS/MS panels, inhibitor concentrations approximated per-donor IC_{50} values or a maximally effective dose (see Supplementary Tables 7–9).

3.Immunofluorescence (IF)

Cells grown on Matrigel-coated plates (Corning Inc. (Corning, NY, USA), 356237) were washed with phosphate-buffered saline (PBS), fixed in 4% paraformaldehyde for 15 min at room temperature. After blocking with 1% bovine serum albumin (BSA) in PBS, samples were incubated overnight at 4 °C with a primary anti-human CD56 (NCAM) antibody (dilution per datasheet). After PBS washes, an Alexa Fluor 488–conjugated secondary antibody (Thermo Fisher Scientific) was applied, and nuclei were counterstained with DAPI. Coverslips were mounted with an antifade medium and imaged on a LSM 980 confocal microscope (Carl Zeiss, Oberkochen, Germany) under identical acquisition settings across samples. Negative controls (no-primary) were included to assess nonspecific signal. (See Supplementary Figs. 4–5.)

4. Flow cytometry (FACS)

Adherent primary adrenocortical cells were detached with trypsin/EDTA, washed, and resuspended in PBS. To minimize nonspecific binding, cells were pre-incubated for 5 min with an anti-human Fc blocking reagent (BioLegend, Cat#564219, RRID:AB_2728082). Cells were stained per manufacturers' instructions with: CD56-FITC (BD Biosciences, Cat#562794, RRID:AB_2737799), CD31-BUV395 (BD Biosciences, Cat#565290, RRID:AB_2739159), CD45-APC (BD Biosciences, Cat#555485, RRID:AB_398600), CD90-BV421 (BioLegend, Cat#328121, RRID:AB_10933261), and 7-AAD viability dye (BioLegend, Cat#420403). After 20–30 min at 4 °C, cells were washed and filtered (40 µm). Acquisition was performed on a BD FACSymphony A3 (BD Biosciences); data were analyzed in FlowJo v10.9.0. Gating: debris exclusion (FSC/SSC) → singlets (FSC-A vs FSC-H) → live cells (7-AAD$^-$) → population annotation: CD56$^+$ adrenocortical cells; CD31$^+$ endothelial, CD45$^+$ lymphocyte, and CD90$^+$

stromal lineages (see Supplementary Figs. 6–8). Unstained/single-stained controls and, where applicable, FMOs supported threshold placement.

4. Immunohistochemistry (IHC) on FFPE tissue

Four-μm sections were processed on Ventana Discovery Ultra (Roche Diagnostics GmbH (Mannheim, Germany)) using EZ Prep and Cell Conditioning 1 (CC1) for heat-induced epitope retrieval, with Inhibitor CM for peroxidase blocking. Primary antibodies: anti-CYP11B1 (rabbit mAb, clone EPR3793, Abcam ab70198, 1:400), anti-CYP11B2 (mouse mAb, 1:800; gift from Dr. Celso E. Gomez-Sanchez), anti-CYP17A1 (rabbit mAb, clone EPR7312, Abcam ab134938, 1:1000), and anti-CD56 (mouse mAb, clone 123C3, Agilent M730429-2). Detection used Discovery OmniMap HRP reagents with ChromoMap DAB; hematoxylin counterstain per instrument protocol. Isotype-matched and no-primary controls were included.

5. Steroid measurements by LC–MS/MS

Comprehensive steroid profiling was performed by liquid chromatography–tandem mass spectrometry (LC–MS/MS) at ASKA Pharmaceutical Medical Co., Ltd. (Tokyo, Japan) to quantify androstenedione (A-dione), corticosterone (B), 11-deoxycorticosterone (11-DOC), 11-deoxycortisol (11-DOF), dehydroepiandrosterone (DHEA), DHEA sulfate (DHEA-S), progesterone, 17-hydroxyprogesterone (17-OHP), and testosterone (T).

6. Concentration–response modeling and statistics

Responses were normalized to vehicle = 100%. Curves were fit to a three-parameter logistic (3PL) model with top fixed at 100% and bottom constrained ≥0; EC_{50} (IC_{50}) and HillSlope were free. Span was defined as the dynamic range (top − bottom), expressed as %. Curves not achieving ≥50% inhibition within 0–10 μM at 72 h were labeled NE (not estimable). IC_{50} values are summarized as geometric means with 95% CIs (asymmetric profile-likelihood). Between-

drug comparisons used a mixed-effects model on log(IC_{50}) (donor as a random effect) with Tukey adjustment. Multi-group endpoints, including span, used Kruskal–Wallis with Dunn post hoc tests. Two-sided $\alpha = .05$. Analyses were performed in GraphPad Prism v10.4 and R v4.01.

7. Molecular docking

Receptors: human CYP11B2 (PDB ID: 4zgx) and CYP11B1 (PDB ID: 6m7x) models were built from published crystal structures. Waters/ions were retained or removed per original annotations; heme parameters and axial ligation were retained as in the PDB files. Docking: AutoDock Vina (v1.2.5) with grid boxes centered on the catalytic heme. The simulation was run with the following parameters: cpu = 8, exhaustiveness = 8, num_modes = 20, and energy_range = 3. Scoring/inspection: Vina scores (kcal/mol). Top-ranked poses were visualized in UCSF ChimeraX and inspected for catalytic geometry (Fe–ligand distance/angle, steroid orientation) and steric compatibility.

8. Quality control and exclusion criteria

Experiments with baseline viability <60% or contamination were excluded a priori. Analytical outliers were resolved by blinded re-integration; biological outliers were retained unless pre-specified criteria were met (e.g., non-monotonic response across ≥3 adjacent concentrations).

9. Reagents and software

Key reagents, media, antibodies, and software used in this study are summarized below.

This table consolidates IF/FACS and IHC reagents, media/buffers, platforms, and software into a single list. Where information is not available, "—" is used. Please replace any TBD entries with final catalog/lot information.

Category	Item / Target	Fluor /	Application	Vendor	Catalog #

		Type			
Enzyme	Collagenase Type I	—	Tissue dissociation	Worthington	LS004194
Enzyme	Collagenase Type VII	—	Tissue dissociation	Worthington	LS005332
Buffer	ACK Lysing Buffer	—	RBC lysis	Gibco	A1049201
Matrix	Matrigel, growth factor–reduced	—	IF (coverslip coating)	Corning	356237
Medium	DMEM/F12	—	Base medium	Gibco	11320033
Serum	Fetal bovine serum (FBS)	—	Supplement	Gibco	A5670201
Antibiotic	Penicillin–Streptomycin (100×)	—	Antibiotic supplement	Gibco	15140122
Enzyme reagent	Trypsin/EDTA (0.05%)	—	FACS (cell detachment)	Gibco	25300062
Buffer	PBS	—	IF/FACS (wash/resuspension)	Gibco	10010023
Fixative	Paraformaldehyde (4%)	—	IF (fixation)	Nakacai	09154-85

Blocking	BSA (1% in PBS)	—	IF (blocking)	Thermo Scientific	37525
Antibody (IF primary)	Anti-human CD56 (NCAM)	FITC	IF (primary)	BD	562794
Nuclear stain	DAPI	—	IF (nuclear stain)	VEC	H-1200
Antibody (IHC)	CYP11B1 (clone EPR3793)	—	IHC (primary)	Abcam	ab70198
Antibody (IHC)	CYP11B2 (mouse mAb)	—	IHC (primary)	—	—
Antibody (IHC)	CYP17A1 (clone EPR7312)	—	IHC (primary)	Abcam	ab134938
Antibody (IHC)	CD56 (mouse mAb 123C3)	—	IHC (primary)	Agilent	M730429-2
Platform (IHC)	Ventana Discovery Ultra	—	IHC platform	Roche	—
Blocking	Anti-human Fc block	—	FACS (block)	BioLegend	564219
Antibody (FACS)	CD56 (NCAM)	FITC	FACS (marker)	BD Biosciences	562794

Antibody (FACS)	CD31	BUV395	FACS (lineage)	BD Biosciences	565290
Antibody (FACS)	CD45	APC	FACS (lineage)	BD Biosciences	555485
Antibody (FACS)	CD90 (Thy-1)	BV421	FACS (lineage)	BioLegend	328121
Viability dye	7-AAD	—	FACS (viability)	BioLegend	420403
Software	AutoDock Vina (v1.2.5)	—	Docking	Scripps Research	—
Software	UCSF ChimeraX (v1.5)	—	Visualization	UCSF	—

Abbreviations: BSA, bovine serum albumin; FACS, fluorescence-activated cell sorting; IF, immunofluorescence; IHC, immunohistochemistry; PBS, phosphate-buffered saline; RT, room temperature; TBD, to be determined.

10. Data and code availability

Raw and processed data, analysis scripts (R/Prism), and docking input files (PDBQT, grid parameter files) are available upon reasonable request to the corresponding author.

Supplementary Table 1. Proportion of each cell type in Human Primary Adrenocortical Culture cells

Patient No.	Percentage in live cells (%)			
	CD56	CD31	CD45	CD90
**Aldosterone-producing adenoma**				
R004	38.8	3.4	NT	NT
A005	45.7	3.3	12.0	19.0
A009	46.6	2.6	1.5	13.7
A011	47.5	21.5	8.6	0.8
A013	29.5	2.7	0.3	36.5
A018	18.8	14.7	2.6	30.9
**Cortisol-producing tumor**				
C003	35.1	32.5	9.5	8.5
C004	20.2	21.4	7.2	38.8
C005	64.8	14.9	5.9	2.9
C015	22.6	41.1	21.0	0.8
C016	9.8	38.8	2.5	44.1
C019	45.9	22.2	11.8	9.1
C020	7.3	2.3	0.1	45.3
C021	50.7	11.5	4.8	12.0
**Normal adrenal gland**				
N001	32.4	2.5	0.5	46.7

Abbreviations: NT, Not tested

Supplementary Table 2. Bottom and Top values of dose-dependent curve of Baxdrostat, Osilodrostat, and Metyrapone for Aldosterone and Cortisol Production by Human Primary Adrenocortical Cultures

		Bottom (%)			Top (%)		
Patient No.	Symbol	Baxdrostat	Osilodrostat	Metyrapone	Baxdrostat	Osilodrostat	Metyrapone
**Aldosterone-producing adenoma (for Aldosterone)**							
A005	●	33.7 (17.3 - 48.4)	44.0 (NE – 87.5)	51.2 (NE – 92.3)	120.3 (107.5 - 133.8)	104.8 (72.4 – 139.9)	99.9 (58.4 – 142.1)
A009	■	8.8 (NE - 19.8)	9.1 (3.5 – 14.6)	5.3 (NE – 17.1)	101.0 (93.0 - 109.1)	98.2 (92.0 – 104.6)	101.7 (96.5 – 106.9)
A010	▲	12.5 (NE - 31.2)	14.3 (4.6 – 23.8)	NT	113.1 (101.8 - 124.6)	104.2 (94.6 – 114.0)	NT
A011	▼	19.8 (16.4 - 23.1)	19.7 (16.5 – 22.9)	26.5 (0.5 – 46.7)	100.4 (97.5 - 103.3)	100.2 (96.0 – 104.4)	97.2 (89.3 – 105.6)
A013	♦	19.6 (17.4 - 21.8)	NT	NT	100.0 (97.3 - 102.7)	NT	NT
A018	○	8.2 (NE - 18.9)	NT	24.3 (20.0 – 28.5)	93.6 (77.6 - 112.3)	NT	100.0 (97.5 – 102.4)
R001	□	NT	34.5 (21.1 – 47.2)	0.0 (NE – 95.5)	NT	105.8 (95.2 – 116.6)	110.4 (96.0 – 125.2)
R002	⬣	NT	25.9 (1.6 – 47.5)	0.0 (NE – 69.5)	NT	102.1 (75.7 – 130.3)	98.2 (58.4 – 147.8)
R003	◇	NT	24.7 (NE – 55.7)	0.0 (NE – 137.0)	NT	113.6 (83.4 – 145.3)	118.8 (56.6 – 184.9)
R004	⬡	NT	17.4 (NE – 36.0)	0.0 (NE – NE)	NT	100.3 (78.8 – NE)	100.5 (63.0 – 138.8)
**Cortisol-producing tumor (for Cortisol)**							
C003	●	NC	5.4 (NE – 35.8)	0.0 (NE – 20.4)	NC	121.5 (96.7 – 148.0)	107.1 (99.1 – 115.5)
C004	■	NC	6.6 (1.2 – 11.9)	NT	NC	101.8 (97.9 – 105.8)	NT
C005	▲	NC	0.0 (NE – NE)	0.0 (NE – NE)	NC	105.3 (99.5 – 111.0)	103.3 (98.1 – 108.6)
C013	▼	NC	0.0 (NE – 59.6)	0.0 (NE – 39.7)	NC	107.5 (89.2 – 126.6)	104.2 (91.5 – 117.5)
C015	♦	NC	4.5 (NE – 36.0)	NT	NC	94.2 (77.2 – 111.5)	NT
C016	○	NC	5.9 (NE – 26.0)	0.0 (NE – 27.2)	NC	92.8 (81.6 – 104.4)	100.6 (85.1 – 117.0)
C018	□	NC	6.9 (NE – 26.9)	0.0 (NE – 32.6)	NC	103.9 (93.2 – 114.7)	109.4 (97.3 – 121.7)
C019	△	NC	7.1 (NE – 27.0)	NT	NC	91.5 (81.9 – 101.4)	NT
C020	⬣	NT	NT	0.0 (NE – 12.9)	NT	NT	102.2 (97.2 – 107.4)
C021	◇	NC	11.1 (NE – 21.6)	6.7 (NE – 19.5)	NC	96.0 (89.1 – 102.9)	95.3 (88.4 – 102.3)
**Normal adrenal gland (for Cortisol)**							
N003	■	NC	4.3 (NE – 13.8)	0.0 (NE – 12.7)	NC	96.0 (89.6 – 102.6)	102.7 (94.0 – 111.7)
N004	▲	NC	NT	NT	NC	NT	NT

Values are reported with 95% confidence intervals. Abbreviations: NE, Not estimable; NT, Not tested; NC, Not calculable

Supplementary Table 3. Geometric mean IC50 values of each steroid synthase inhibitor for aldosterone production in Human Primary Adrenocortical Cultures of Aldosterone-producing adenoma

drugs	n	GM IC_{50} (μM)	95% CI lower	95% CI upper
**Baxdrostat**	6	0.0407	0.0129	0.1289
**Osilodrostat**	8	0.0022	0.0009	0.0050
**Metyrapone**	8	0.8939	0.4098	1.9498

Abbreviations: GM, Geometric mean; CI, Confidence interval

Supplementary Table 4. Comparison of geometric mean IC50 values of each steroid synthase inhibitor for aldosterone production in Human Primary Adrenocortical Cultures of Aldosterone-producing adenoma

Comparison	Mean difference (log10)	Adjusted p value	GMR
**Baxdrostat vs. Osilodrostat**	1.044 (-0.0418 - 2.1290)	0.0553	11.066 (0.9082 - 134.59)
**Baxdrostat vs. Metyrapone**	-1.473 (-3.1200 - 0.1730)	0.0664	0.0337 (0.0008 - 1.4894)
**Osilodrostat vs. Metyrapone**	-2.741 (-3.0730 - -2.4080)	< 0.0001	0.0018 (0.0008 - 0.0039)

Values are reported with 95% confidence intervals. Comparisons between groups were performed using mixed-effects analysis followed by Tukey's multiple comparison test. Abbreviations: GMR, Geometric mean ratio

Supplementary Table 5. Geometric mean IC50 values of each steroid synthase inhibitor for cortisol production in Human Primary Adrenocortical Cultures of Cortisol-producing tumor

drugs	n	GM IC_{50} (μM)	95% CI lower	95% CI upper
**Baxdrostat**	9	NC	NC	NC
**Osilodrostat**	9	0.1958	0.0720	0.5322
**Metyrapone**	7	0.6290	0.3434	1.1520

Abbreviations: GM, Geometric mean; CI, Confidence interval

Supplementary Table 6. Comparison of geometric mean IC50 values of each steroid synthase inhibitor for cortisol production in Human Primary Adrenocortical Cultures of Cortisol-producing tumor

Comparison	Mean difference (log10)	p value	GMR
**Osilodrostat vs. Metyrapone**	0.4087 (-0.1938 - 1.011)	0.1417	0.3902 (0.0975 - 1.5624)

Values are reported with 95% confidence intervals. Comparisons between groups were performed using paired t test.
Abbreviations: GMR, Geometric mean ratio

Supplementary Table 7. Effect of 1 µM Baxdrostat, Osilodrostat, and Metyrapone for 12 Steroids by Human Primary Adrenocortical Cultures of Aldosterone producing adenoma

Steroid	Percent change (%)		
	Baxdrostat (n = 5)	Osilodrostat (n = 4)	Metyrapone (n = 2)
**Aldosterone-producing adenoma**			
Androstenedione	+50.8 ± 45.9	+354.6 ± 147.8[a]	+214.9 ± 22.4
Corticosterone	-28.4 ± 17.4	-73.6 ± 6.7[a]	-70.0 ± 8.0
11-deoxycorticosterone	+376.4 ± 146.2	+2539.2 ± 720.7[a]	+767.3 ± 206.9
Dehydroepiandrosterone	+12.6 ± 34.5	+3.1 ± 5.3	+10.3 ± 19.8
Progesterone	+0.2 ± 21.4	+16.2 ± 20.5	-2.3 ± 5.0
17-hydroxyprogesterone	-52.1 ± 9.2	-5.0 ± 18.6	+16.3 ± 5.3
Testosterone	+12.4 ± 22.4	+290.3 ± 119.9	+209.2 ± 25.4
Aldosterone	-72.9 ± 3.6	-75.8 ± 4.2	-51.4 ± 18.8[a,b]
Cortisol	-46.0 ± 24.5	-85.0 ± 4.6	-73.5 ± 2.6
Dehydroepiandrosterone-sulfate	-48.7 ± 13.0	-28.0 ± 13.3	+3.3 ± 24.6
11-deoxycortisol	+65.0 ± 26.4	+1571.5 ± 421.5[a]	+1182.9 ± 44.5
18-hydroxycorticosterone	-73.2 ± 5.0	-78.4 ± 4.8	-68.4 ± 13.5

The values are displayed as mean ± SEM.

[a] $P < 0.05$ compared with the percent change of baxdrostat.

[b] $P < 0.05$ compared with the percent change of osilodrostat.

Supplementary Table 8. Effect of 1 µM Baxdrostat, Osilodrostat, and Metyrapone for 12 Steroids by Human Primary Adrenocortical Cultures of Cortisol producing tumor

	Percent change (%)		
**Steroid**	**Baxdrostat (n = 9)**	**Osilodrostat (n = 9)**	**Metyrapone (n = 7)**
**Cortisol-producing tumor**			
Androstenedione	+98.7 ± 47.8	+352.7 ± 160.5	+365.5 ± 108.2
Corticosterone	+6.5 ± 9.8	-77.6 ± 4.4[a]	-65.4 ± 6.8[a]
11-deoxycorticosterone	+159.7 ± 66.0	+1091.7 ± 249.6[b]	+784.1 ± 162.3[d]
Dehydroepiandrosterone	+81.6 ± 51.5	+11.7 ± 14.5	+7.0 ± 9.9
Progesterone	+4.6 ± 20.3	+26.0 ± 16.0	+48.3 ± 8.4
17-hydroxyprogesterone	-13.8 ± 14.3	+2.4 ± 14.2	+37.6 ± 9.2
Testosterone	+86.1 ± 48.4	+375.5 ± 195.5	+337.7 ± 100.2
Aldosterone	-51.0 ± 13.3	-67.8 ± 11.4	-71.9 ± 8.2
Cortisol	-7.3 ± 8.6	-74.2 ± 6.8[a]	-62.1 ± 7.0[a]
Dehydroepiandrosterone-sulfate	+11.2 ± 16.2	-37.5 ± 3.1[c]	-26.7 ± 2.0[d]
11-deoxycortisol	+70.5 ± 24.0	+1205.7 ± 326.3[b]	+1209.6 ± 251.1[c]
18-hydroxycorticosterone	-42.8 ± 15.5	+20.4 ± 57.1	-73.2 ± 19.3

The values are displayed as mean ± SEM.

[a] $P < 0.0001$ compared with the percent change of baxdrostat.

[b] $P < 0.001$ compared with the percent change of baxdrostat.

[c] $P < 0.01$ compared with the percent change of baxdrostat.

[d] $P < 0.05$ compared with the percent change of baxdrostat.

Supplementary Table 9. Effect of 1 µM Baxdrostat, Osilodrostat, and Metyrapone for 12 Steroids by Human Primary Adrenocortical Cultures of Normal adrenal gland

	Percent change (%)		
**Steroid**	**Baxdrostat (n = 3)**	**Osilodrostat (n = 2)**	**Metyrapone (n = 1)**
**Normal adrenal gland**			
Androstenedione	+5.7 ± 5.5	+684.5 ± 472.9	+263.1
Corticosterone	-16.9 ± 7.7	-89.9 ± 1.8	-88.1
11-deoxycorticosterone	+10.4 ± 11.5	+696.3 ± 30.3	+423.8
Dehydroepiandrosterone	-3.1 ± 1.7	+15.6 ± 3.3	+8.0
Progesterone	-37.2 ± 10.0	+180.1 ± 99.8	+19.1
17-hydroxyprogesterone	-11.4 ± 8.5	+66.0 ± 8.0	+5.8
Testosterone	+6.4 ± 2.9	+1545.7 ± 1289.6	+281.9
Aldosterone	-76.8 ± 11.2	-88.2 ± 4.5	-63.1
Cortisol	-20.7 ± 11.2	-90.3 ± 0.9	-89.0
Dehydroepiandrosterone-sulfate	+6.9 ± 4.4	-17.4 ± 0.2	-16.5
11-deoxycortisol	+2.5 ± 6.0	+1916.6 ± 812.2	+1704.5
18-hydroxycorticosterone	-44.1 ± 19.9	-99.4 ± 0.6	-100.0

The values are displayed as mean ± SEM.

Supplementary Table 10. Clinical and experimental data of additional validation samples

Patient No.	Sex	Clinical Diagnosis	Age at Surgery (years)	Blood Pressure (mmHg)	PRA (ng/mL/hr)	PAC (pmol/L)	ACTH (pmol/L)	F (nmol/L)	Serum Potassium level (mmoL/L)	F (nmol/L) after 1 mg Dexamethasone suppression test	Tumor side	Size of Lesion (cm)	Pathological Diagnosis	Weiss Score
**<u>Aldosterone-producing adenoma</u>**														
A019	Female	PA	62	143/103	0.5	549.3	6.4	311.8	3	46.9	Left	1.4×0.8×1.3	adrenocortical adenoma	0
A021	Female	PA	49	139/90	<0.2	1045.8	8.3	416.6	2.9	30.3	Right	1.6×1.4×2.3	adrenocortical adenoma	0
**<u>Cortisol-producing tumor</u>**														
C028	Female	SCS	54	149/98	0.3	139.0	<0.3	281.4	3.8	361.4	Right	1.6×3.1×2.8	adrenocortical adenoma	1
C029	Female	SCS	67	136/76	0.5	121.8	<0.3	480.1	3.9	441.4	Left	2.5×2.3×2.4	adrenocortical adenoma	0

Tissue diagnosis was based on the pathology report. Blood pressure is indicated as systolic/diastolic blood pressure. Plasma ACTH, PAC, and F values were converted to SI units using the following factors: ACTH (1 pg/mL = 0.2202 pmol/L), PAC (1pg/mL = 2.774pmol/L), and F (1μg/dL = 27.59nmol/L). Abbreviations: PRA, Plasma renin activity; PAC, Plasma aldosterone concentration; ACTH, Adrenocorticotropic hormone; F, Cortisol; PA, Primary aldosteronism; SCS, Subclinical Cushing's syndrome
